# HBV genome-enriched single cell sequencing revealed heterogeneity in HBV-driven hepatocellular carcinoma (HCC)

**DOI:** 10.1186/s12920-022-01264-2

**Published:** 2022-06-16

**Authors:** Wenhui Wang, Yan Chen, Liang Wu, Yi Zhang, Seungyeul Yoo, Quan Chen, Shiping Liu, Yong Hou, Xiao-ping Chen, Qian Chen, Jun Zhu

**Affiliations:** 1 Department of Genetics and Genomic Sciences, Icahn School of Medicine at Mount Sinai, 1425 Madison Ave., New York, NY 10029 USA; 2 Icahn Institute for Genomics and Multiscale Biology, Icahn School of Medicine at Mount Sinai, New York, NY USA; 3 Sema4, Stamford, CT USA; 4 The Hepatic Surgery Centre at Tongji Hospital, Tongji Medical College, Huazhong University of Science and Technology (HUST), Wuhan, China; 5 BGI, Shenzhen, China; 6 Department of Mathematics, Hebei University of Science and Technology, Shijiazhuang, Hebei China; 7 The Division of Gastroenterology, Department of Internal Medicine at Tongji Hospital, Tongji Medical College, Huazhong University of Science and Technology (HUST), Wuhan, China; 8 The Tisch Cancer Institute, Icahn School of Medicine at Mount Sinai, New York, NY USA

**Keywords:** Hepatocellular carcinoma, Hepatitis B virus integration, Enriched single cell sequencing, Copy number variation, Clonal evolution

## Abstract

**Background:**

Hepatitis B virus (HBV) related hepatocellular carcinoma (HCC) is heterogeneous and frequently contains multifocal tumors, but how the multifocal tumors relate to each other in terms of HBV integration and other genomic patterns is not clear.

**Methods:**

To interrogate heterogeneity of HBV-HCC, we developed a HBV genome enriched single cell sequencing (HGE-scSeq) procedure and a computational method to identify HBV integration sites and infer DNA copy number variations (CNVs).

**Results:**

We performed HGE-scSeq on 269 cells from four tumor sites and two tumor thrombi of a HBV-HCC patient. HBV integrations were identified in 142 out of 269 (53%) cells sequenced, and were enriched in two HBV integration hotspots chr1:34,397,059 (*CSMD2*) and chr8:118,557,327 (*MED30/EXT1*). There were also 162 rare integration sites. HBV integration sites were enriched in DNA fragile sites and sequences around HBV integration sites were enriched for microhomologous sequences between human and HBV genomes. CNVs were inferred for each individual cell and cells were grouped into four clonal groups based on their CNVs. Cells in different clonal groups had different degrees of HBV integration heterogeneity. All of 269 cells carried chromosome 1q amplification, a recurrent feature of HCC tumors, suggesting that 1q amplification occurred before HBV integration events in this case study. Further, we performed simulation studies to demonstrate that the sequential events (HBV infecting transformed cells) could result in the observed phenotype with biologically reasonable parameters.

**Conclusion:**

Our HGE-scSeq data reveals high heterogeneity of HCC tumor cells in terms of both HBV integrations and CNVs. There were two HBV integration hotspots across cells, and cells from multiple tumor sites shared some HBV integration and CNV patterns.

**Supplementary Information:**

The online version contains supplementary material available at 10.1186/s12920-022-01264-2.

## Background

Hepatocellular carcinoma (HCC) is ranked as the third most lethal cancer worldwide [1], and 54% of HCC cases originate from chronic Hepatitis B Virus (HBV) infection [2]. During HBV infection, a small fraction of viral replication is in double-stranded linear DNA form, which can be inserted into the host genome at double-stranded break points [3]. HBV integrations only occur in the early phase of HBV infection [3, 4]. HBV integration into the human genome is one of the most important etiological mechanisms of HBV induced HCC [5]. Recurrent HBV integrations have been identified by sequencing studies [6–11].

HBV-HCC tumors are of high heterogeneity in terms of HBV DNA integration patterns and somatic genomic alterations, and this heterogeneity is associated with prognosis and drug response in HBV-HCC [12]. Both empirical and simulation studies show that only integration events of high allele frequency can be detected at a given sequencing depth [9, 13]. In general, it is expensive to implement whole genome sequencing (WGS) with high sequencing depth in a large scale study. HIVID (high-throughput Viral Integration Detection) by Li et al. [14] provides an efficient way to accurately detect HBV integration in the whole genome. Regions containing virus genome sequences are enriched in the process of preparing the DNA library such that the genomic regions to be sequenced for identifying virus integration sites are remarkably smaller than the whole human genome. Recently, HIVID has been applied in sequencing of a large number of HBV-HCC samples [15] as well as in detecting Human papillomavirus (HPV) integration sites [16].

DNA single cell sequencing has demonstrated its power in studying tumor clonal expansion and tumor heterogeneity. Navin et al*.* [17] first introduced DNA single cell sequencing techniques in tumor evolution studies. In the study, although only 6% of the genome was covered due to limitations of the whole genome amplification technique (Sigma-Aldrich GenomePlex WGA4 kit), computational methods were developed to accurately estimate DNA copy number variations (CNVs). Zong et al*.* [18] proposed the multiple annealing and looping-based amplification cycles (MALBAC) for whole genome sequencing.

Both GenomePlex and MALBAC are extensively reviewed and compared with multiple displacement amplification (MDA) under different circumstances [19–26] due to the vital importance of Whole Genome Amplification (WGA) in DNA single cell sequencing. However, no WGA method is reliable in all situations. Some studies suggest MDA as the best overall approach [20, 21, 23] while others disagree [19]. In general, studies [24–26] indicate that MDA performs well in terms of single-nucleotide variations detection and CNVs detection. Single cell sequencing has been used in studying human brain cells [27], kidney cancer [28], lung cancer [29], bladder cancer [30], JAK2-negative myeloproliferative neoplasm [31], and gamete genomes of individuals [32]. More recently, Wang et al*.* [33] and Leung et al*.* [34] significantly improved the WGA technique by sequencing cells in the G2/M stage when cellular DNA content is duplicated compared to other stages. By doing so, the coverage width is increased to 91%, making it possible to study the single nucleotide variation at the single cell level [33, 34].

As single cell sequencing technology advances, several open questions about HBV-HCC tumorigenesis need to be re-examined. (1) What is the frequency of HBV integration? The frequency of HBV integration is estimated in the range of 1 in per 1000 hepatocytes [35, 36]. The expected frequency of two HBV integrations in one hepatocyte is ~ 10^–6^, an unlikely event under a normal condition as suggested in literature [3]. As HBV integrations occur in the early phase of HBV infection [3, 4], HBV integrations will not increase during tumorigenesis. Thus, multiple HBV integrations occurring in one hepatoma cell is highly unlikely as well. However, there are HBV-HCC cell lines with multiple integrations [37, 38]. A single cell genome sequencing study also indicates that there are 5–6 HBV integrations in a cell, which are also identified by bulk tissue WGS [39]. It has been shown that tumor-initiating cells are more prone to HBV integration due to genome instability [40]. It is then possible that integration frequency is much higher in cells prone to double-stranded breaks [41]. (2) What is the role of HBV integrations, initiating tumorigenesis or accelerating clonal expansion of tumor-initiating cells? (3) How are multifocal HBV-HCC tumors related in terms of HBV integrations and CNV patterns?

To address these questions, we present an approach based on HBV genome-enriched single cell sequencing (HGE-scSeq) to identify the heterogeneity of HBV integrations and genomic alterations in HBV-HCC tumor cells at the single cell level. We performed HGE-scSeq on cells from four independent tumor sites and two tumor thrombi from a HBV-HCC patient (Additional file 16: Fig. S1). In addition, we performed a series of simulation studies to evaluate whether sequential events can result in observed phenotypes within biologically reasonable parameters.

## Methods

### Patient and tissue samples

The study of tumor cell heterogeneity was approved by an Institutional Review Board (IRB) at Tongji Hospital, Tongji Medical College of HUST, in Hubei province, China (IRB #TJ-C20111217). The signed written informed consent was obtained before patient’s recruitment, according to the regulations of the institutional ethics review boards. The patient and sample information (Additional file 16: Fig. S1) was detailed in Chen et al. [42]. The clinicopathological information of the patient is summarized in Additional file 2: Fig. Table S1.

### HBV genome enriched single cell sequencing (HGE-scSeq)

The fresh (within one hour after surgery) frozen (stored in − 80 °C) tumor tissue samples were thawed in a water bath at room temperature and digested into cell solution by collagenase as previously described [31]. With sufficient collagenase dissociation and dilution, the cancer tissues were separated into single cells solution, cell clusters and cell debris. Massive cell clusters were filtered out when the suspensions were injected into a membrane filter (pore size = 20 µm). To avoid contamination with cell debris, suspensions were then re-suspended and centrifuged in Phosphate Buffered Saline (PBS) five times. After filtration, cell suspensions were added into a PBS droplet containing 0.5% BSA. Single cell isolation was performed using a micropipette as previously described [31] under microscope and cells with intact cell membranes were randomly selected for single cell sequencing.

For each cell, WGA was performed with MDA using REPLI-g Mini Kit (QIAGEN, Inc.) according to the instructions of the manufacturer as previously described [31]. HIVID [14] procedure was then used to enrich sequences containing HBV genome sequence. The DNA library from the amplified single cell genome was hybridized with the biotinylated HBV probe to enrich DNA fragments containing HBV DNA sequences. Then, the enriched libraries were quantified and subjected to 101 cycles paired-end index sequencing in Illumina HiSeq 2000 sequencer according to manufacturer's instructions (Illumina Inc., San Diego, CA). The raw data are deposited at NIH SRA (BioProject: PRJNA553308).

### Mapping HGE-scSeq reads

On average, 17.39M (17,393,993) reads were generated for each cell. Low quality reads were filtered out according to the following criteria. If any single read in a read pair had more than half base of quality less than five, the corresponding read pair was filtered (Additional file 17: Fig. S2A). If a read pair was contaminated by adaptor sequences, it was filtered. If two read pairs were the same, only one copy was kept in further analysis. After quality filtration, 5.49M (5,494,183) reads were kept in further analyses. Among them, 77.13% and 0.24% were aligned to the human and HBV genomes, respectively, on average. With paired-end assembly and re-mapping, reads supporting virus integration were identified (Additional file 17: Fig. S2B, detailed below). The number of reads supporting HBV virus integration in each cell was in a range of 0 to 53,290. The average percentage of human genome covered by sequencing reads was 3.13% with an average depth of coverage 3.14, which was used to estimate CNVs (Additional file 17: Fig. S2C, detailed below). The detailed information of reads distribution can be found in Additional file 3: Table S2 and Additional file 4: Table S3.

### Bulk tissue HBV enriched DNA sequencing

Corresponding adjacent non-neoplastic liver tissues for the four independent tumor sites, noted as N1-4, were collected for bulk tissue HBV enriched DNA sequencing. For the four adjacent normal tissues, the HIVID procedure was directly applied to the extracted DNA without the WGA step, followed by the same 101 cycles paired-end index sequencing. On average, 45.96M reads were generated for each tissue sample. After quality filtration 12.13M reads were kept for further analyses. Among them, 78.48% reads were mapped to the human genome, and 0.013% reads were mapped to the HBV genome. On average, only 50 reads supporting HBV integration were detected for each control tissue sample. The average percentage of human genome covered by reads was 6.9% with average depth of coverage 1.272. The detailed information of reads distribution can be found in Additional file 3: Table S2 and Additional file 4: Table S3.

### Quality check of whole genome sequencing reads

Our previously described pipeline [13] was used to process the whole genome sequencing data. In brief, prinseq-lite [43] was used to filter the reads that were exactly the same or of mean reads quality lower than 20 and more than 10% Ns. The remaining reads were mapped to the human genome with Bowtie2 (version: 2.2.8 -D 15 -R 2 -N 0 -L 22 -i S,1,1.15) [44]. Duplicated reads after alignment were filtered using Picard (version: 2.2.4).

### Quality check of chimera reads in HGE-scSeq data

Limited amount of input material from a single cell for WGA causes a lot of technical errors, including low physical coverage, non-uniform coverage, allelic dropout events, false positive and false negative errors due to insufficient coverage [18–21, 23, 26, 33, 45, 46]. Chimera reads, which can be partially mapped to different parts of the genome that are not physically linked [26], are common artifacts of single cell WGA [26], which can interfere with our ability to identify HBV-human genome chimera sequences. The frequency of chimera reads (identified following the standard protocol [26, 47]) was 0.025%. Also the number of chimera reads from both inter-chromosome and intra-chromosome were independent from the number of HBV-Human soft clipped reads, HBV reads and Human reads (Additional file 18: Fig. S3).

### Quality check of reads mapped to human genome

A large fraction of sequencing reads was mapped to the human genome even though the regions containing HBV sequences were enriched in the sequencing library preparation step. To check whether loci covered by sequencing reads were randomly distributed across the human genome, for each locus, we counted the number of cells with reads covering the locus. If the reads mapped to the human genome were randomly distributed, then the number of cells with reads at each locus is expected to follow a Poisson distribution. The largest number of cells with reads covering a locus was 209, the mean was 8.2277, and the fraction of loci not covered by reads in any cell was 11.22% (Additional file 19: Fig. S4). The observed distribution was tested against a Poisson distribution with a chi square test on range of [1, k] (k indicates a locus covered by reads in k cells, which corresponds to the kth bar in Additional file 19: Fig. S4) with k from 15 to 37 (Additional file 5: Table S4). The distribution matched with a Poisson distribution until k = 28, which corresponds to 87.97% of the human genome. When k ≥ 29, the distribution was no longer a Poisson distribution. Thus, the mapped reads on the majority of the human genome follow a Poisson distribution, except the region consistently missed by all cells and 0.81% of the human genome covered by reads from a number of cells significantly more than expected by chance. These observations suggested that a CNV profile at single cell level can be accurately estimated with the appropriate normalization method.

### Comparing human genome regions with and without HGE-scSeq reads

To infer CNVs from reads mapped to the human genome, these reads should be evenly distributed across the human genome and there should be no systematic difference between the regions covered with sequencing reads and the regions without. To investigate the property of the regions with and without read sequence coverage, we first constructed a Fisher machine prediction model [48] to distinguish HBV and human genome sequences by randomly sampling 10,000 sequences of 100 bp length from HBV and human genomes. Then, we applied the Fisher machine to test whether the sequences in the human genome regions with or without HGE-scSeq reads were similar to HBV or human genome sequences. For each cell, 10,000 sequences of 100 bp length were randomly sampled from human genome regions with and without mapped reads, and input them to the Fisher machine. There was no difference between scores of regions with and without mapped reads (Wilcox rank sum test p-value = 0.3636, Additional file 20: Fig. S5).

### Mapping to HBV virus genomes

The filtered reads were aligned to UCSC hg19 with soap2 [49] (Version: 2.20) in paired-end mode (Additional file 17: Fig. S2B). The parameters used were “-s 85 -l 50 -v 2 –r 1 -p 6 -m 100 –x 500”. If any read in a pair was not mapped to the human genome, the pair was kept as a candidate for virus detection. These reads were collected and transformed from FASTQ to FASTA format. The virus detection part in VirusFinder [50, 51] was used to detect the virus. The reads not mapped to the human genome were aligned to a virus database, which contains the genomes of all known viruses (32,102 in total) [52]. The reads aligned to a virus genome were de novo assembled into contigs. Then, the contigs were aligned to the human genome and virus database. The contigs that could be aligned to the human genome were filtered out. If the percentage of identity between the contig and virus’ genome was less than 85% or less than 75% of the contig was aligned to a reference genome, the alignment was filtered out. The alignment score of contigs was defined as the multiplication of the mapped length of the contig and percentage of identity between the mapped region of the contig and the virus genome. The virus substrains were ranked by the maximum alignment score of contigs aligned to its genome. The top ranked virus substrain was reported as the matched virus substrain in the cell (Additional file 6: Table S5). The top common substrains were all HBV B subtypes and were similar in sequences (Additional file 7: Table S6).

### Detecting HBV integration sites

The reads not mapped to the human genome were aligned to the detected virus genome using soap2 (Version: 2.20 with the following parameters “-s 85 -l 50 -v 5 –r 1 -p 6 -m 100 -x 500”). The paired-end reads not mapped to the human genome or virus genome were collected and assembled to long reads using flash (version: 1.2.11 with parameters “-m 5 × 0.2 –p 64”) [53]. The designed smaller insertion size compared to the total length of a pair of reads enabled most read pairs to be assembled into one read of much longer length. The assembled reads were aligned to the human genome and virus genome using bwa and bwasw [54] (version: 0.7.15 -a 1 –b 2 –q 5 –r 2). The soft clipped reads with at least 30 bp aligned to the human genome and at least 30 bp aligned to the virus genome were collected for identifying the integration sites. If the distance between two breakpoints was less than 20 bp on both the human genome and HBV genome, we defined them as one breakpoint which was supported by reads combined from the two breakpoints. In order to make the predicted integration events between different cells comparable, we also merged integration sites within 20 bp when collecting the predicted integration sites across different cells. The number of soft clipped reads was tightly correlated with the number of HBV reads (Additional file 18: Fig. S3F). We normalized soft clipped reads against the number of HBV reads. The optimal threshold of soft clipped reads for HBV integration was selected to minimize the correlation between numbers of HBV reads and detected HBV integrations as detailed in Additional file 1: Methods.

### Estimating CNVs

Reads mapped to the human genome were randomly distributed (Additional file 19: Fig. S4), which enabled us to estimate DNA copy numbers across the human genome. Because the sequencing data was based on an enriched single cell sequencing protocol [14], the existing pipelines for detecting CNVs in single cell sequencing data [17, 55, 56] were not directly applicable. If applied directly, more regions of copy number aberration than the regions of normal copy number would be identified, which is counter intuitive. Therefore, a new pipeline for inferring CNVs was developed for analyzing the data set (detailed in Additional file 1: Methods).

### Evaluation of read count correction

Sequences containing HBV sequence were enriched at the DNA library preparation step. To correct and evaluate read count bias due to enrichment sequencing, we assessed read dispersion using two matrices as detailed in Additional file 1: Methods.

### Evaluating the CNV pipeline with reads from normal control

Our CNV pipeline was modified from a CNV pipeline for single cell sequencing data, which takes full consideration of correcting for bias incorporated from WGA [57]. Evaluation of our modified pipeline on bulk tissue enrichment sequencing data is detailed in Additional file 1: Methods.

### Association between clone evolution and HBV integrations

A parsimony method is mostly recommended for constructing phylogenetic trees from single cell CNV profiles [58, 59]. Therefore, in this study, we used a parsimony method [58] to build phylogenetic trees based on CNVs at the 49 identified CNV segments (detailed in Additional file 1: Methods).

## Results

### HCC patient and tissue information

**Fig. 1:**
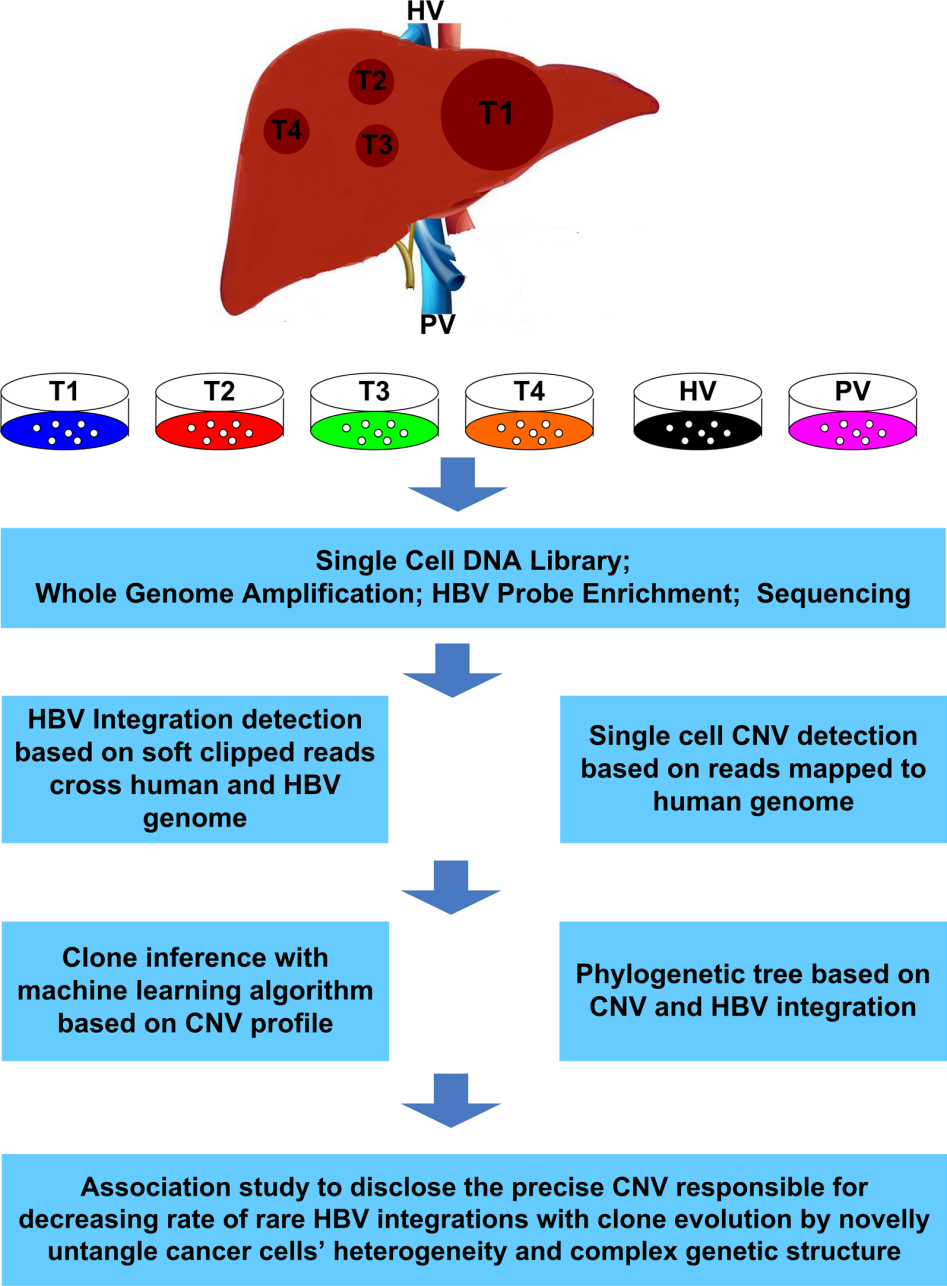
Overview of the study. 269 cells from four tumor tissues and two thrombi tissues were extracted. HBV genome sequence enrichment was performed after whole genome amplification on the single cell DNA genome. Pair-end sequencing was used. A pipeline was developed for HBV integration identification and CNV inference. Tumor clones were inferred based CNV profile. Association between HBV integration and CNV was assessed based on clone inference and phylogenetic tree. Key CNVs differentiate two clones were identified with phylogenetic tree. Statistical test was performed on the key genetic regions while considering only cells belonging to related clones. Images in the figure are drew by the authors

The study was approved by an Institutional Review Board (IRB) (detailed in “Methods”) and was conducted according to the principles of the Declaration of Helsinki. A middle-aged (between 40- to 50-year-old) patient matched with the research design. Obtained medical history indicated that the patient had no history of alcohol abuse, recognized acute hepatitis, mother-to-child transmission of HBV, blood transfusion, or injection drug use. Tests indicated the patient had a resolved HBV infection (HBs Ab level 884.5 mIU/mL, HBs Ag-negative, HBc Ab-positive, HBe Ab-positive, HCV Ab-negative, and blood HBV undetectable). MRI revealed a 15 cm × 10 cm main lesion in the left hepatic lobe and multiple smaller lesions in the right hepatic lobe, all under 3 cm in diameter (Additional file 16: Fig. S1A). Tumor thrombi involved in the right portal vein branch (PVTT) and inferior vena cava (IVCTT) were revealed by MRI with contrast enhancement, indicating the intrahepatic and extrahepatic vascular spreading of HCC (Additional file 16: Fig. S1B). Tumor was TNM stage IV and surgical resection was performed. Tumor tissues from the 4 tumor sites (noted as T1–4) and corresponding adjacent normal tissues as well as tissues from two tumor thrombi were collected after surgery. Additional information can be obtained from the “Methods” section. To understand inter- and intra-tumor heterogeneity at the single cell level, we designed the study as outlined in Fig. 1.

### Identification of HBV integration sites and estimation of CNVs in HBV-HCC cell line MHCC97H

MHCC97 is a HBV positive, highly metastatic HCC cell line [60]. MHCC97H is further isolated from MHCC97 due to its higher metastatic potential [61]. We characterized MHCC97H by WGS with 1,485,306,632 100 bp pair-end reads. After read QC [13] (“Methods”), 1,308,162,600 reads were mapped to the human genome with average 42.2 folds coverage. CNVs of MHCC97H were estimated based on the WGS data. Read counts were normalized and corrected for GC content. Circular binary segmentation (CBS) [62] was used to infer the segmentation. CNVs of MHCC97H were also measured using SNP arrays (GSE38326 [63]). The copy number amplifications based on WGS and SNP arrays were similar (correlation γ = 0.96, Additional file 8: Table S7, Additional file 21: Fig. S6).

We performed HGE-scSeq on five MHCC97H cells. For each cell, 32,253,536 (in average) reads were generated (Additional file 3: Table S2). After read QC (“Methods”), 10,336,455 (in average) reads were included in further analysis. Among them, 19,717 (in average) contained sequences in the HBV genome, and 5,452,432 (in average) were mapped to the human genome (Additional file 3: Table S2).

#### HBV integration sites of MHCC97H

For the WGS data, we applied the pipeline as described previously [13] and set the threshold of supporting reads (one soft clipped read or two adjacent reads). Total five HBV integration sites were identified (Additional file 9: Table S8). For HGE-scSeq data, between 22 and 69 integration sites were identified in each cell, resulting in a total of 176 unique integration sites (“Methods”, Additional file 10: Table S9). When considering WGS and HGE-scSeq data derived integration sites that were within 5000 bp of each other as the same site, 57 of the HBV integration sites based on single cell data matched with four integration sites based on WGS (Highlighted in Additional file 10: Table S9). Each cell had two-four integrations common with the integrations identified by WGS. Among 176 HBV integration sites, 41 were identified in at least two cells (Additional file 10: Table S9).

#### CNV estimations of MHCC97H

Even though sequencing libraries were enriched for HBV genome sequences, an average of 52.97% of reads were mapped to the human genome and 2.68% of the human genome covered with at least one read. Some regions were covered by multiple reads. Numbers of reads at each locus across the human genome followed a Poisson distribution (Additional file 22: Fig. S7, chi-sq test, p-value 0.98). And the loci covered by reads in multiple cells were enriched in copy number amplified regions defined by WGS (Additional file 23: Fig. S8). To check whether there were any genome feature differences between human genome regions with and without mapped reads, we first constructed a Fisher machine prediction model [48] to distinguish HBV and human genomes (Additional file 24: Fig. S9A, “Methods”). Then, we applied the Fisher machine to quantify sequence feature differences between genome regions with and without mapped reads. There was no clear difference between human genome regions with and without mapped reads (Additional file 24: Fig. S9B&C). These results together suggest that HGE-scSeq reads were dispersed randomly across the human genome.

We developed a method to infer CNVs based on HGE-scSeq data (“Methods”) and applied it to infer CNVs of the MHCC97H cell line. The inferred CNVs based on HGE-scSeq data were consistent with WGS and SNP array data (correlation γ = 0.85–0.92 and 0.8–0.88, respectively, Additional file 8: Table S7 and Additional file 21: Fig. S6).

#### Heterogeneity of MHCC97H cells

A single cell genomic sequencing study of HepG2 cells suggests that HepG2 cells are heterogeneous in term of CNVs [64], and the variation of CNVs among cells are consistent with transcription level variations at the CNV regions, suggesting the variations are unlikely due to random errors in single cell sequencing. Our HGE-scSeq data of MHCC97H cells identified common HBV integrations and revealed heterogeneity in terms of both HBV integrations and CNVs at the single cell level.

### HGE-scSeq of multifocal HBV-HCC tumors

**Fig. 2:**
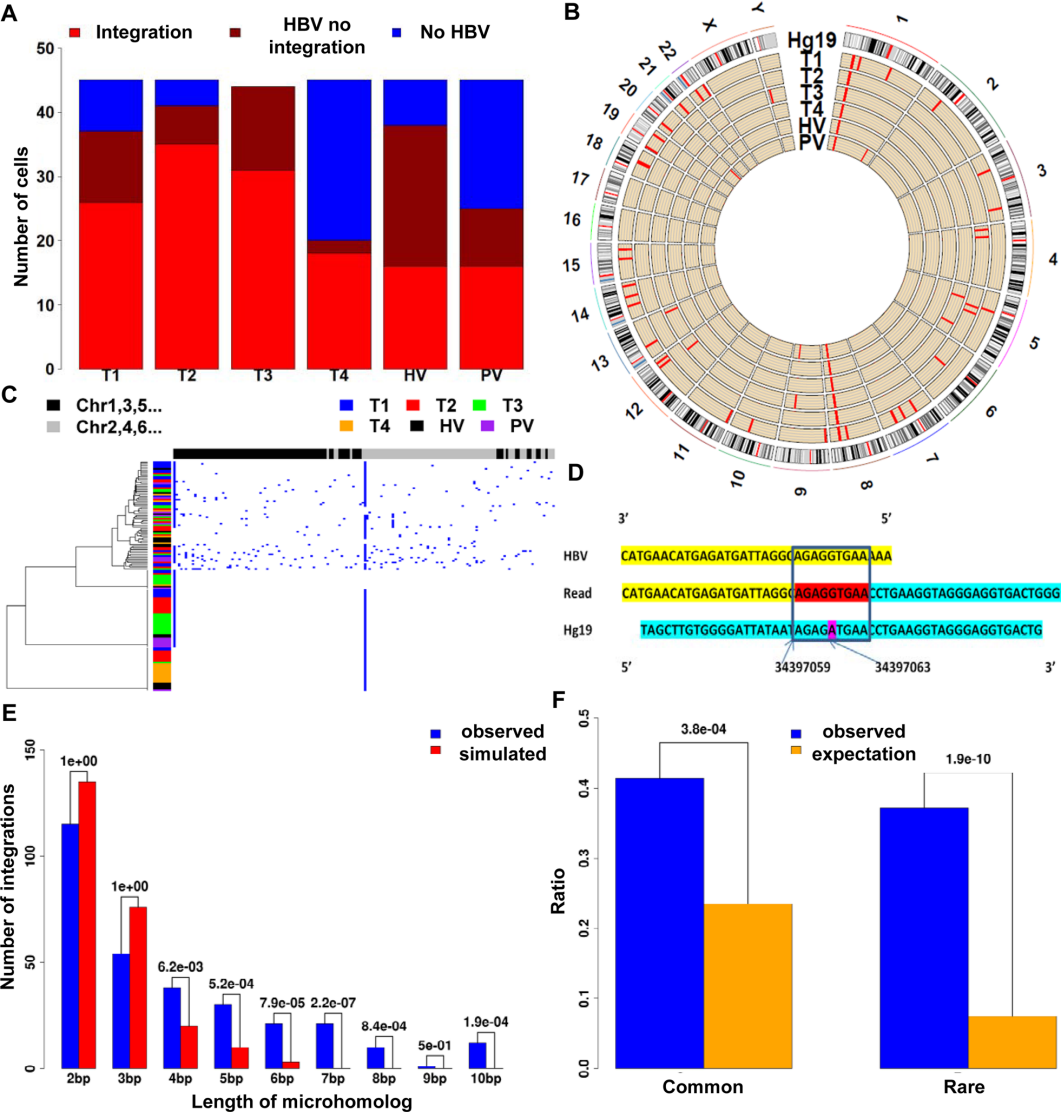
HBV integration heterogeneity and mechanisms of HBV integration. **A** Fractions of cells in each tissue with or without HBV sequences detected. **B** Circos map of integration; each circle indicates integrations identified in a tumor tissue. **C** HBV integration distribution across the human genome. Each row represents the integration profile of a cell. The cells are labeled by its tissue source. The columns are loci with HBV integrations along chromosomes. The cells were clustered by hierarchical clustering. **D** An example of Microhomolog between sequences of the human genome and HBV genome at an HBV integration hotspot site Chr1 34,307,059. There are two 4 bp homologs between human genome and HBV genome (AGAG and TGAA) with 1 bp mismatch in the middle. **E** Microhomology enrichment. Numbers of HBV integrations carrying different length of homology sequences between human genome and HBV genome near the HBV integration sites were collected (blue). The observed numbers were significantly different from the numbers based on random simulations (red). **F** Fragile region enrichment. Both common and rare fragile regions on the human genome were enriched for HBV integrations

HGE-scSeq was applied to 269 cells from 6 sites (Additional file 16: Fig. S1). HBV virus sequence reads were detected in 205 out of the 269 cells (detailed in “Methods”, Fig. 2A). HBV assemblies were close to HBV isolate G247-B3 (an example of pileup of sequencing reads is shown in Additional file 25: Fig. S10). It is worth noting that HBV sequencing reads from normal tissues contained reads covering the whole HBV genome (Additional file 25: Fig. S10A). In contrast, the HBV virus assemblies from all single cells missed most of the HBV genomic region encoded for X protein (Additional file 25: Fig. S10B).

#### Heterogeneity of HBV integrations

Before identifying HBV integration sites, chimera reads were examined. Numbers of inter chromosome and intra chromosome chimera reads were correlated, and they both correlated with the length of chromosome, consistent with random nature of human chimera reads (Additional file 26: Fig. S11). The number of soft clipped reads and the number of HBV reads were strongly correlated (Additional file 18: Fig. S3F), which suggests that the number of HBV reads is needed to be considered when identifying HBV integration.

Among the 205 cells with HBV sequence reads detected, HBV integrations were detected in 142 cells (detailed in “Methods”). A total 471 integration events were identified (Additional file 11: Table S10, which corresponds to 164 unique integration sites (Additional file 12: Table S11). The HBV integration sites were not evenly distributed across the human genome (Fig. 2B). There were two integration hotspots, chr1: 34,397,059 (*CSMD2*) and chr8:118,557,327 (*MED30/EXT1*), where the integration events were identified in 100 and 121 cells, respectively (Fig. 2B). With regard to HBV genome, most of HBV integrations located in HBVgp2_S, HBVgp3_X and HBVgp4_Precore/Core proteins (Additional file 27: Fig. S12A) with the integrations at the hotspot on human chr1 mapped to HBVgp3_X while the ones at the hotspot on chr8 mapped to HBVgp4_Precore/Core. The distribution of HBV integration sites across the HBV genome is shown in Additional file 27: Fig. S12B. On average 3.32 integration events were detected in each cell. Based on the HBV integration profile, cells were clustered into two groups with one group only carrying integrations at the hotspots and the second group carrying extra rare integrations (Fig. 2C). Numbers of sequencing reads for cells in the two groups were similar (Additional file 28: Fig. S13). Most integration sites were detected only in one cell. Only 39 integration sites were detected in multiple cells or multiple tumor sites. The heterogeneity on frequency of HBV integrations across cells and tissues was observed. All the cells with HBV integration carried at least one of the hotspot integrations. The HBV integration sites were distributed across 46 genes or gene pairs based on UCSC known genes. The integration sites at the two hotspots, chr1: 34,397,059 (*CSMD2*) and chr8:118,557,327 (*MED30/EXT1*), were not reported in previous HBV integration studies (except in this dataset as we previously reported [42]), but overlapped with multiple fusion events from both cancer cell lines and TCGA [65] (Additional file 13: Table S12).

Next, we compared HBV integration patterns in adjacent normal tissues close to the four tumor sites. In total, 17 integration events (Additional file 11: Table S10) were detected at 13 loci (Additional file 12: Table S11) in the four adjacent normal tissues. The numbers of HBV integrations in adjacent normal tissues and in tumors were not directly comparable as one based on bulk tissue sequencing and one based on single cell genomic sequencing. In a loose sense, there were more integration events in tumors than in normal tissues than tumors, consistent with previous reports [15]. The integration sites at the two hotspots were also detected in each adjacent normal tissue except that the integration site at chr1 hotspot was not detected in N1 and chr8 hotspot integration was not detected in N2 (in which only one soft clipped read was detected and less than the minimum threshold of two soft clipped reads). The integration events at the two hotspots were the only two recurrent events across four adjacent normal tissues. The available information is not sufficient to distinguish whether HBV integrations at the two hotspots in adjacent normal tissues were results of clone expansion or diffusion from tumor tissues. Additional information is needed to inform clonal relationships between cells with HBV integrations at the two hotspots in adjacent normal and tumor tissues.

#### Properties of HBV integration sites

Based on single cell sequencing data we identified 164 unique integration sites. Micro-homologous sequences between the human genome and HBV genome (an example shown in Fig. 2D) were enriched at the HBV integration sites (Fig. 2E). We also found the enrichment of integration sites within the common and rare fragile regions [66] (Fig. 2F). The enrichment of micro-homologous sequences near HBV integration and enrichment of HBV integration on fragile regions elucidate that the HBV integration is a physical driven process, which is highly related with the sequence content and corresponding physical characteristics of host genome sequence.

#### HBV integration hotspots

The two integration hotspots, chr1: 34,397,059 and chr8:118,557,327 are located at the intronic region of *CSMD2* and the intergenic region of *MED30-EXT1*, respectively. The chr1 hotspot could partially be explained by microhomology (Fig. 2D), which led to loss of *CSMD2* expression. The integration at the chr8 hotspot resulted in over-expression of *EXT1*, which promoted cell growth in vitro and in vivo [42].

#### Heterogeneity of CNVs

**Fig. 3:**
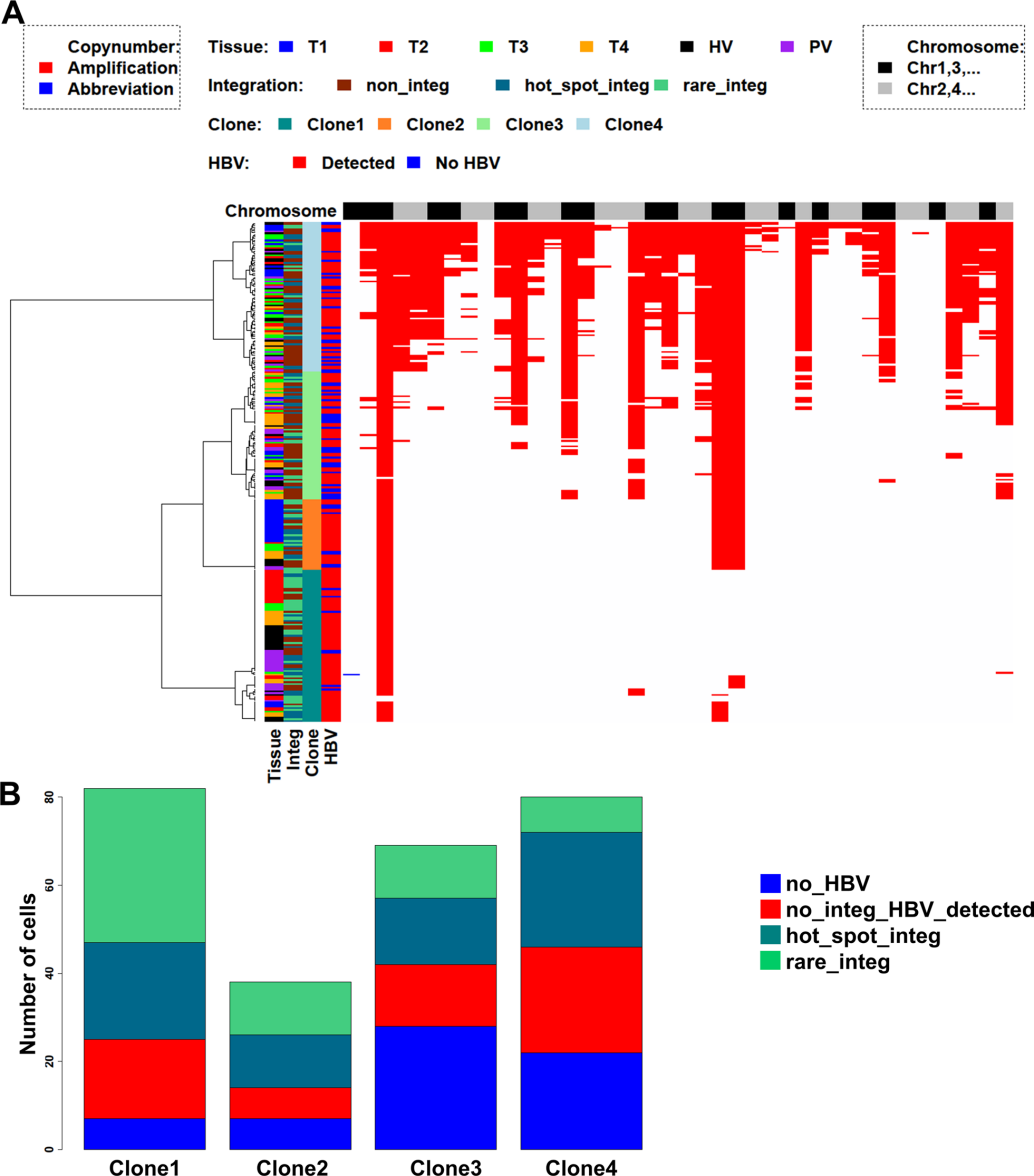
CNV heterogeneity at single cell level. **A** CNV profiles. Each row corresponds to a cell. The cells are labeled with regard to source tissue, clone annotation, HBV integration category and HBV sequence detection result. Each column corresponds to a bin. The bins are ordered by their chromosome locations (chromosome 1–22). Cells can be categorized into 4 groups corresponding to 4 clones. White means normal copy number, blue indicates copy number loss, red indicates copy number amplification. **B** Composition of cells with no HBV detected, cells with HBV sequence detected but no integration, cells carrying rare integration, and cells carrying only hotspot integration only in each clone. The frequency of cells carrying rare HBV integrations is highest for clone 1 and lowest for clone 4. The frequencies for clones 2 and 3 were comparable, and both were lower than the one for clone 1

In addition to HBV integration, we estimated each cell’s CNVs based on the HGE-scSeq data (“Methods”). As expected, most of the bins had a normal copy number of DNA (Fig. 3A). All cells carried a DNA copy number amplification at chromosome 1q, which is a recurrent feature of HCC [67] (Fig. 3A). The cells were clustered into 4 clone groups based on CNVs (Fig. 3A), each clone had a distinct pattern of DNA copy number amplifications. And each clone group contained cells with different types of HBV integrations (Fig. 3B). From clones 1 to 4, the ratio of cells carrying rare integrations decreased.

#### Clonal evolution and its relationship with HBV integration

**Fig. 4:**
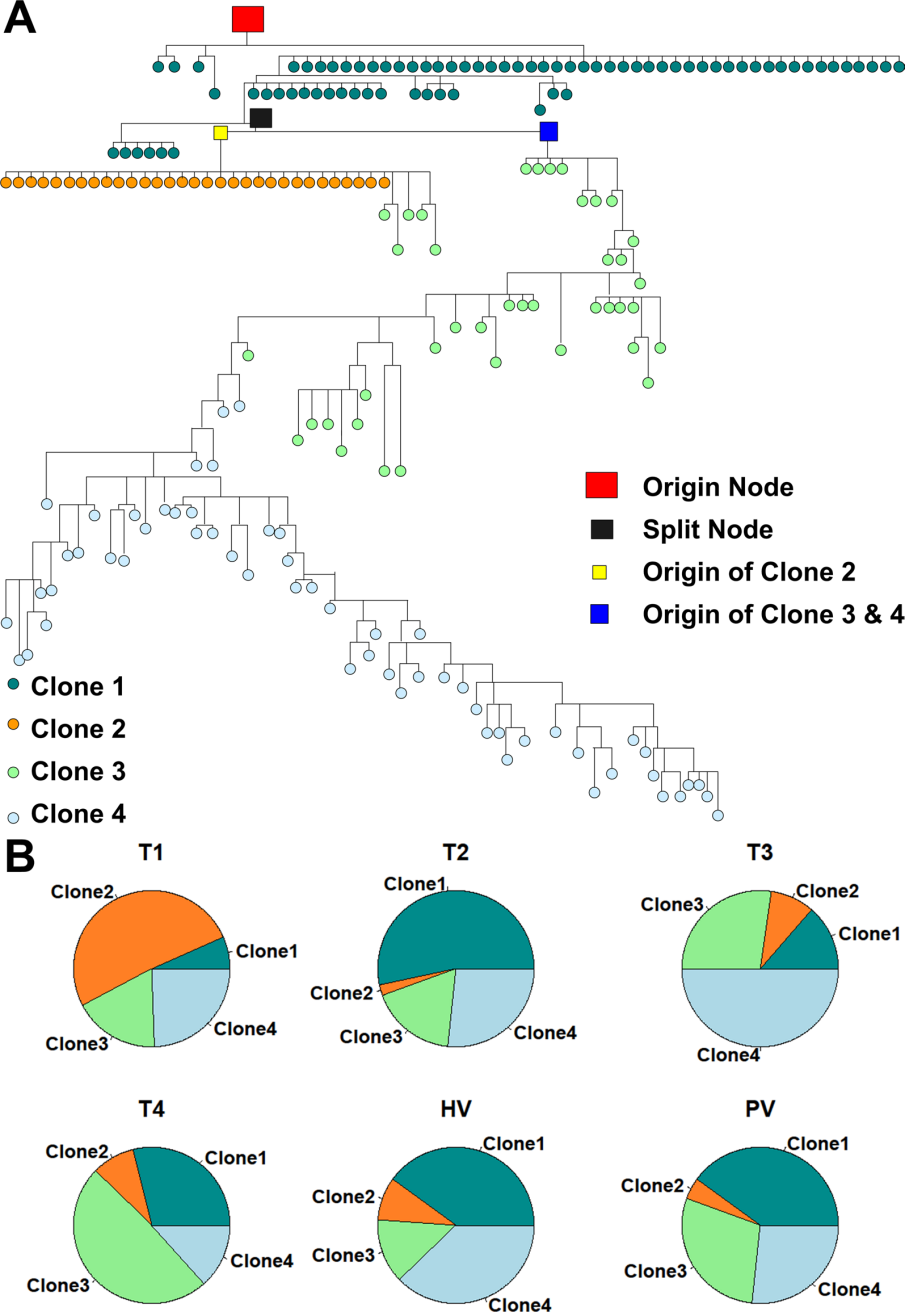
Clonal relationship of cells from different tumor sites. **A** A phylogenetic tree built based on single cell CNV profiles. Each node corresponds to a cell. The cells are colored according clone annotation. Splitting nodes are marked as squared nodes. The scale of splitting node correlates to the number of its decedent nodes. **B** Clone composition of each tumor tissue**.** Pie plots for each bin on the fractions of four clones. Each tumor tissue had one major clone and three minor clones. There was no single major clone in the thrombus tissues, but clones 3 and 4 together accounted for more than 50% of cells in the tumor thrombi, suggesting the two clones were more invasive

Based on the CNV pattern, we constructed a phylogenetic tree (detailed in “Methods”, Fig. 4A), which suggests that clone 1 directly developed from the ancestor. Clone 2 and clones 3&4 were derived from clone 1, suggesting there were two different evolution directions. The inner node corresponding to the origin of clone 2 and clones 3&4 as well as the inner node corresponding to the split between clone 2 and clones 3&4 were annotated in Fig. 4A. These inner nodes can be directly linked to CNVs on a specified region. The root node in the phylogenetic tree corresponded to the cells with CNVs of 1q. The common origin of clones 2, 3, and 4 had Chr11 amplification. The regions differentiating clones 2–4 from clone 1 contained potential genomic regions that may associate with the decreasing ratio of rare integration carrying cells. Cells in clone 2 contained CNVs on Chr11 while cells in clones 3 and 4 contained additional CNVs at Chr8:118,268,000–146,364,000. More CNVs split clones 3 and 4. Additional file 29: Fig. S14 is the same as Figs. 4A, except nodes colored according to cells with hotspot and rare HBV integrations. It is clear that rare HBV integrations were not randomly distributed in the phylogenetic trees.

To identify the potential CNV regions associated with decreasing number of rare HBV integrations, we tested the association between CNV and HBV integrations in the clone evolution process from clone 1 to clones 2–4 and the split between clone 2 and clones 3&4 separately. The significant regions (Additional file 14: Table S13) associated with the HBV integration difference between clone 1 vs. clones 2–4 were enriched for immune related genes (Table 1). Genes encoding for secretoglobin family proteins (*SCGB1A1*, S*CGB1D1*, *SCGB1D2*, *SCGB1D4*, *SCGB2A1*, and *SCGB2A2*) were enriched in the regions (Fold change = 50.9, p-value = 5.8E−8). Secretoglobin family 1 proteins have anti-inflammation and immunomodulation property [68] and are inducible by interferon-gamma [69]. Members (*APOA1*, *APOA4*, *APOA5*, *SAA1*, *SAA2*, and *SAA4*) of high density liporprotein (HDL) were significantly enriched in the regions (Fold change = 32, p-value = 8.5E−7). It has been shown that serum HDL level is reversely associated with serum HBV DNA level [70]. Similarly, the AIM2 inflammasome complex was enriched (p-value = 2.8E−5, Fold change = 58.4), which contains genes *CASP1*, *CASP4*, *CASP4* and *CASP12*. In addition, *AIM2* is located in chromosome 1q, which was amplified in all cells (Fig. 3). The AIM2 inflammasome complex is reported contributing to the defense against bacterial and double-stranded viral DNA [71]. Another annotated inflammasome IPAF complex was enriched (p-value = 1.4E−5, Fold change = 70.1). Inflammasomes have been shown to relate to both cancer suppression and promotion under different contexts, which makes them a double-edged sword for cancer development [72]. Serum amyloid A (SAA) proteins, which were also significantly enriched in the regions (Fold change = 115.6, p-value = 2.4E−6), interact with inflammasomes [73]. For the evolution process separating clone 2 and clone 3&4, significant regions consisting of 48 genes (Additional file 15: Table S14) were identified. These genes were enriched for genes in the Urokinase-type plasminogen activator receptor (uPAR) complex (p-value = 1.1E−6, Fold change = 182.4, Table 2), which shows elevated expression during inflammation and tissue remodeling [74], again suggesting that tumor cells of different genomic features may have different capability against HBV replication and HBV insertion. Also, uPAR expression is associated with invasiveness of malignant tumor cells [75], which is consistent with the observation that more than 50% cells in the two tumor thrombi were clones 3 and 4 (Fig. 4B).

**Table 1:** Functional enrichment of genes in the CNV blocks that were significantly different between clone1 and clones 2–4

Category	Term	overlap Genes	Fold Enrichment	P-value	FDR
INTERPRO	Secretoglobin	SCGB1A1,SCGB1D1,SCGB1D2, SCGB1D4,SCGB2A1,SCGB2A2	50.9	5.8E−8	8E−5
UP_KEYWORDS	HDL	APOA1,APOA4,APOA5, SAA1,SAA2,SAA4	32	8.5E−7	1.1E−3
SMART	CARD	CASP1, CASP4, CASP5, CARD16, CARD17	48.2	2.3E−6	2.5E−3
SMART	SAA	SAA2-SAA4, SAA1, SAA2, SAA4	115.6	2.4E−6	2.6E−3
GOTERM_CC_DIRECT	High-density lipoprotein particle	APOA1,APOA4,APOA5, SAA1,SAA2,SAA4	23.9	4.1E−6	5.2E−3
INTERPRO	Serum amyloid A protein	SAA2-SAA4, SAA1, SAA2, SAA4	93.3	4.7E−6	6.6E−3
INTERPRO	Caspase Recruitment	CASP1, CASP12, CASP4, CASP5, CARD16, CARD17	19.3	1.3E−5	1.8E−2
GOTERM_CC_DIRECT	IPAF inflammasome complex	CASP1, CASP12, CASP4, CASP5	70.1	1.4E−5	1.8E−2
GOTERM_CC_DIRECT	AIM2 inflammasome complex	CASP1, CASP12, CASP4, CASP5	58.4	2.8E−5	3.6E−2
GOTERM_MF_DIRECT	Sodium-independent organic anion transmembrane transporter activity	SLC22A11, SLC22A12, SLC22A6, SLC22A8, SLCO2B1	22.2	6.5E−5	8.9E−2
GOTERM_CC_DIRECT	NLRP3 inflammasome complex	CASP1, CASP12, CASP4, CASP5	43.8	7.8E−5	9.9E−2
GOTERM_BP_DIRECT	Sodium-independent organic anion transport	SLC22A11, SLC22A12, SLC22A6, SLC22A8, SLCO2B1	20.1	9.9E−5	1.5E−1
GOTERM_BP_DIRECT	Regulation of apoptotic process	ALX4, CD3E, CASP1, CASP12, CASP4, CASP5, CARD16, CARD17, RPS3, ROBO4, TP53AIP1	4.8	1.1E−4	1.7E−1
SMART	CASc	CASP1, CASP12, CASP4, CASP5	35.6	1.6E−4	1.8E−1

A total of 370 genes were in the regions. DAVID(43) was used to test functional enrichment

**Table 2:** Functional enrichment of genes in the CNV blocks that were significantly different between clone 2 and clones 3 and 4

Category	Term	# overlap Genes	Fold Enrichment	P-value	FDR
UP_SEQ_FEATURE	domain:UPAR/Ly6	LYPD2, LY6K, PSCA, SLURP1	182.4	1.1E−6	1.3E−3
INTERPRO	Ly-6 antigen/uPA receptor -like	LYPD2, PSCA, SLURP1	163.8	1.3E−4	1.3E−1

A total of 48 genes were in the regions. DAVID is used to test functional enrichment

#### Clone 2 vs. other clones

Somatic mutation patterns were derived from bulk tissue whole genome sequencing of T1-4 tumors, two thrombi against the germline genotype based on blood [42]. A phylogenetic tree was constructed based on the somatic mutation patterns, which suggested that T1, the largest tumor, was the primary tumor and other tumors were derived from T1 [42]. Even though all tumors were from the same origin, the clonal composition of each tumor was different. The proportion of clone 2 cells was significantly higher in T1 than in the other tumors (Fig. 4B). To identify differences between clone 2 cells and other cells, we compared CNVs across all bins and identified 282 bins (consisting of 2246 genes) where clone 2 cells had lower CNVs compared to cells of other clones. These genes were enriched in the GO term calcium-dependent cell–cell adhesion (p-value = 9.6 × 10^–7^, Fold enrichment = 3.7, Table 3) and chemokine activity (p-value = 6.2 × 10^–6^, Fold enrichment = 4.0, Table 3). N-cadherin promotes cancer cell invasion [76]. Chemokines and their receptors are involved in tumor immunogenicity and aggressiveness [77, 78]. Lower abundance of chemokines and their receptors might lead to lower potential to metastasis, which may explain why the fraction of clone 2 cells in the primary tumor T1 was higher than the fractions in other tumors (Fig. 4B).

**Table 3:** GO enrichment of genes in the CNV bins where cells of clone 2 had consistently lower CNVs 855 than clones 1, 3, and 4 cells

Category	Term	Genes	P value	Fold enrichment
GOTERM_BP	Calcium-dependent cell–cell adhesion	BCAR1, CDH15, CDH17, CDH2, PCDHB10, PCDHB11, PCDHB13, PCDHB14, PCDHB16, PCDHB2, PCDHB3, PCDHB4, PCDHB5, PCDHB6, PCDHB7, PCDHB8, PCDHB9, YES1	9.6E−07	3.7
GOTERM_MF	Chemokine activity	CXCL1, CXCL2, CXCL3, CXCL5, CXCL6, CXCL9, CXCL10, CXCL11, FAM19A3, IL8, PF4, PF4V1, PPBP, SDF2	6.2E−06	4.0

### Simulation of clonal evolution

**Fig. 5:**
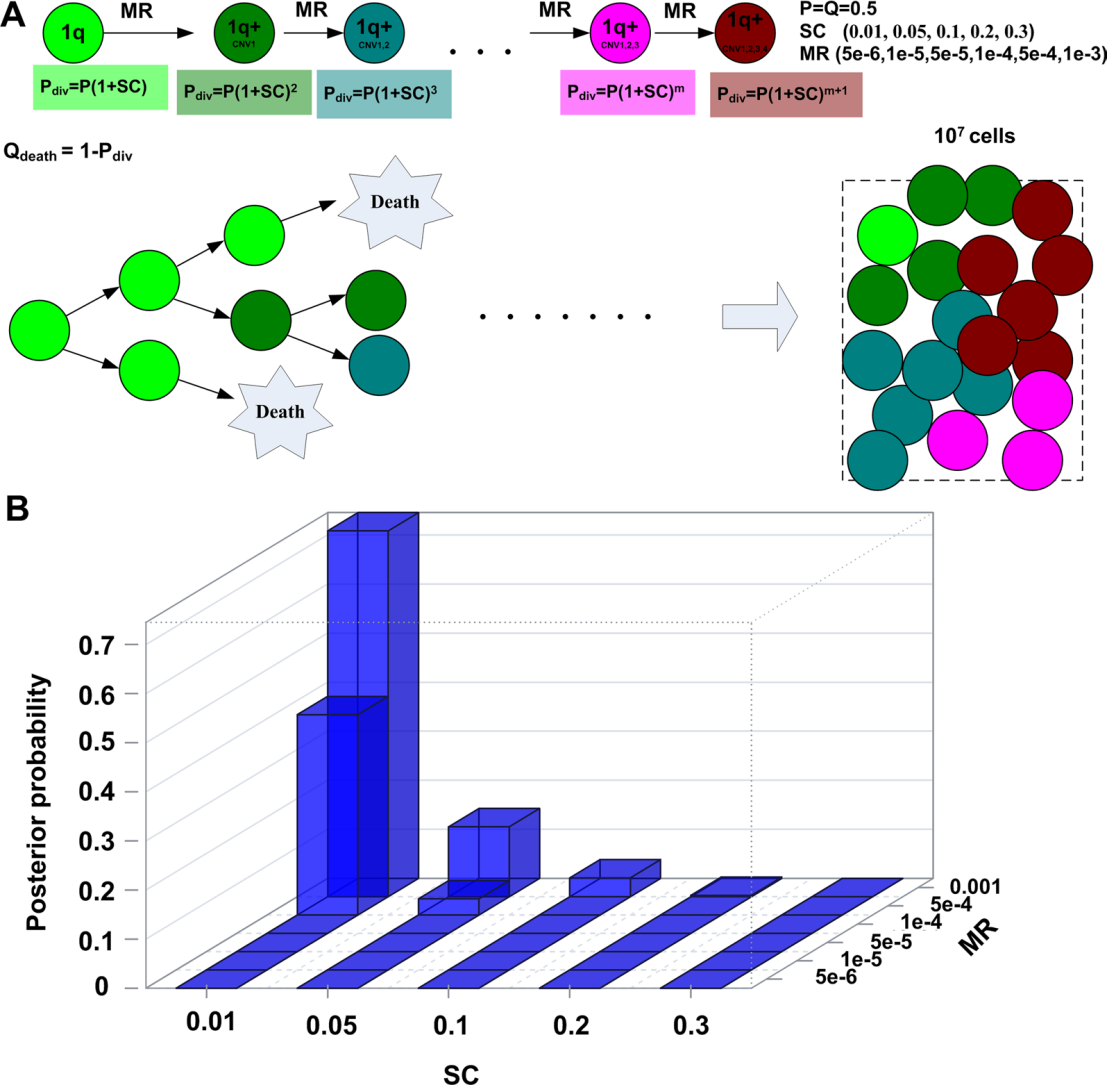
Simulation of clonal evolution with only CNVs. **A** The scheme of birth–death clonal evolution model. Cells accumulated CNVs during cell growth. Each additional CNV increased cell’s probability to divide over to die. **B** Cell populations/tumors were simulated with different combinations of mutation rates (MRs) and selection coefficients (SCs). The posterior probability of each parameter combination was calculated

To assess different clonal evolution scenarios, we performed cell simulations according to the birth–death model [79, 80]. We tested a wide range of parameter space, then calculated the posterior of parameters based on the distance of simulated distribution and the observed data. A simulation starts from a cell after malignant transformation. In the observed data (Fig. 4A), the root node had to carry the chromosome 1q amplification. Otherwise, no simulation resulted in the scenario that 100% cells carried the chromosome 1q amplification. In each replication cycle, a cell divided or died at the probability P_div_ and Q_death_, respectively (Fig. 5A). The simulations stopped when the total number of cells reached 10^7^, corresponding to a tumor of size 0.5 cm × 0.5 cm × 0.5 cm. First, we simulated clonal evolution due to CNV changes without HBV integrations. Each novel CNV change likely alters the fitness of the cell and increases the probability of cell division over the probability of cell death, and the selection coefficient was noted as SC (Fig. 5A). With n number of CNVs acquired in addition to the root event, the division probability was P(1 + SC)^n^, and the corresponding death probability was 1- P(1 + SC)^n^. In a normal cell, the DNA copy number mutation rate (MR) per cell per division is in the range of 10^–10^ to 3.4*10^–6^ [81]. We simulated HCC cells with the copy number mutation rate (5e−6, 1e−5, 5e−5, 1e−4, 5e−4, 1e−3) and the selection coefficient (0.01, 0.05, 0.1, 0.2, 0.3) for each additional CNV. For each simulation, a CNV among the CNVs in Fig. 3A was randomly drawn and introduced to the cell according to the mutation rate. With 10,000 cell populations simulated and compared with the observed one, the posterior of parameters (Fig. 5B) indicated the parameter combination SC = 0.01 and MR = 0.001 fitted the observation the best.

**Fig. 6:**
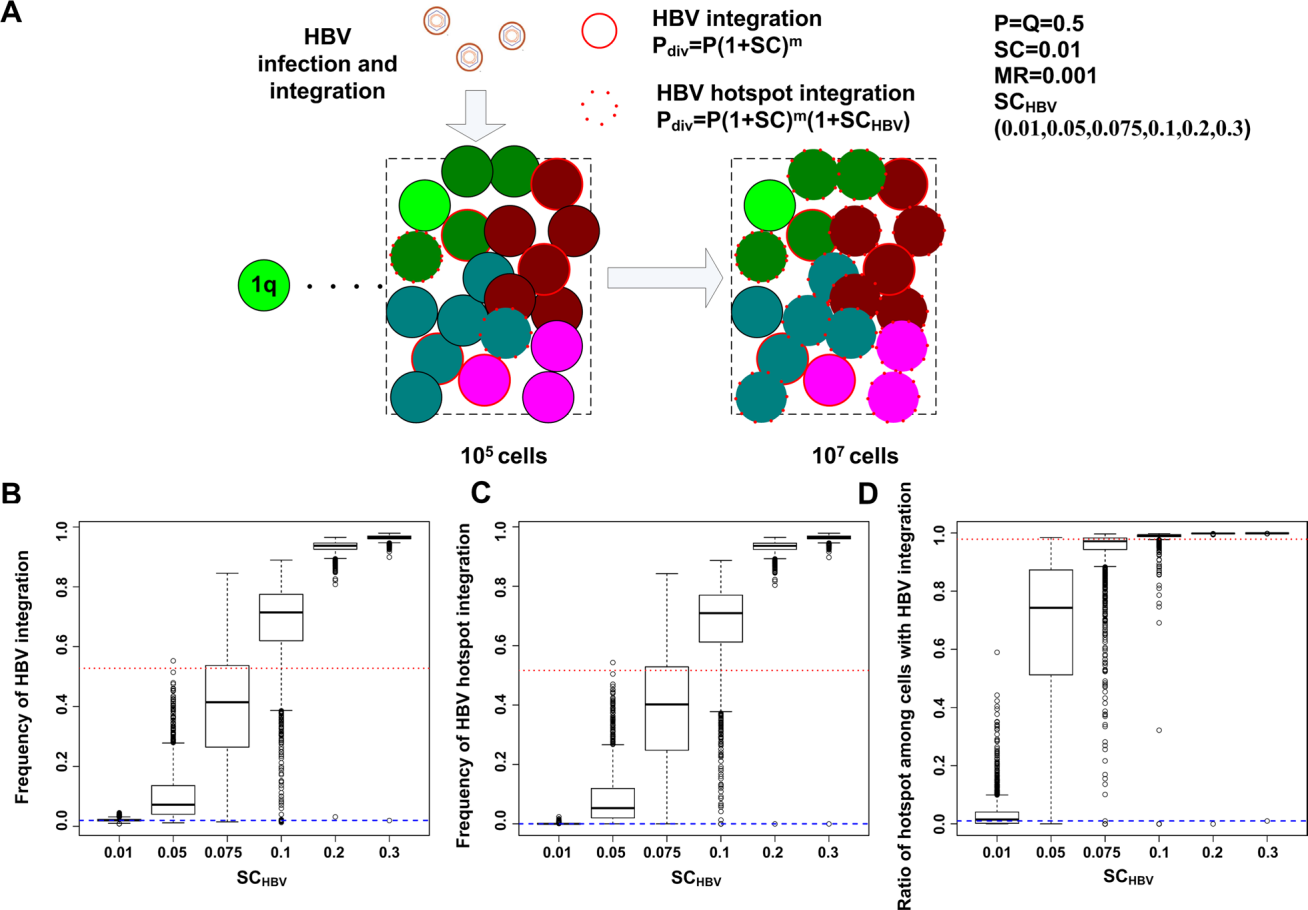
Simulation of clonal evolution with both CNVs and HBV integrations. **A** The scheme of birth–death clonal evolution model with HBV integration. At the tumor size of 10^5^ cells, cells were infected with HBV and HBV integration events occurred. Simulations were generated with the selection coefficient of the hotspot integrations SC_HBV_ in a wide range. **B** The frequency of cells with HBV integrations in the simulated cell populations. The red line is the observed frequency of cells with HBV integrations in the patient data (142/269) and the blue line marks the initial frequency of HBV integration (2%). **C** The frequency of cells with the hotspot HBV integrations in the simulated cell populations. The red line is the observed frequency of cells with the hotspot HBV integrations in the patient data (139/269) and the blue line marks the initial frequency of the hotspot HBV integrations (0.02%). **D** The ratio of cells with the hotspot HBV integrations versus cells with HBV integrations. The red line is the observed ratio in the patient data (139/142) and the blue line marks the initial ratio (1%).

Next, we performed simulations to examine HBV integrations with the parameter combination for CNVs fixed as SC = 0.01 and MR = 0.001 estimated above. We assumed HBV infection occurred when the tumor grew to 10^5^ cells and random HBV integrations occurred in 1 out of 50 HCC cells in the tumor (Fig. 6A). Among the HBV integrations, 1% were hotspot integrations, and only cells with hotspot integrations gained cell growth advantage with the selection coefficient SC_HBV_ in (0.01,0.05, 0.075,0.1,0.2,0.3). Same as above, the simulations stopped when the total number of cells in the tumor reached 10^7^ cells. For each SC_HBV,_ we simulated 2000 cell populations/tumors. Then, we compared the ratios of cells with HBV integrations among cells in tumors at the end of simulation (Fig. 6B). After HBV acute infection, 2% of cells in the simulated tumor carried HBV integrations (blue line in Fig. 6B). When the simulated tumors reached 10^7^ cells, around 50% of cells carried HBV integrations with SC_HBV_ in the range between 0.075 and 0.1, close to the ratio 53% observed in the patient data (red line in Fig. 5B). Similarly, after HBV acute infection, 2 × 10^–4^ of cells in the simulated tumor carried HBV integrations (blue line in Fig. 6C). When the simulated tumors reached a size of10^7^ cells, around 50% of cells carried hotspot HBV integrations with SC_HBV_ in the range between 0.075 and 0.1, close to the ratio 52% observed in the patient data (red line in Fig. 6C), indicating the ratios of cells with hotspot HBV integrations vs. cells with HBV integrations were close to 1 (Fig. 6D).

## Discussion

HBV genome-enriched single cell sequencing approach can efficiently identify HBV integration sites and genomic alterations in HCC cells. We developed a data analysis pipeline for HBV genome enriched single cell sequencing data. Our analyses reveal both highly recurrent and rare HBV integrations in HCC cells. Specifically, a large number of rare HBV integrations were identified in the single cell sequencing study, and these rare HBV integrations suggest that the HBV genome was randomly integrated at sites according to physical properties (Figs. 2E&2F). The mechanism of how the HBV genome is integrated into the human genome is still under-explored. Hu et al. [16] observed significant enrichment of microhomologous sequences at or near 120 HBV integration sites detected from 31 liver samples from Sung et al.[8]. Recently, Zhao et al. [82] sequenced 426 HBV-HCC patients and showed enrichment of micro-homologous sequences around the HBV integration sites as well. Both literature reported observations in bulk tissues and our observations at the single cell level suggest the potential involvement of microhomology mediated mechanisms in the process of HBV integration.

The HBV integration frequency reported here was much higher than one integration expected per 1000 liver cells [35, 36], suggesting that cells with genome instability (leading to higher HBV integration frequency [40]) existed before HBV infection, which is consistent with the observation that all tumor cells had 1q amplification but not all tumor cells had HBV integrations. Upon simulation studies performed here, the event sequence, which is defined as tumorigenesis previous to HBV-infection, can occur if biologically favorable conditions are given (Fig. 6).

There were two HBV integration hotspots (Fig. 2C). The integration hotspot chr1: 34,397,059 (*CSMD2*) could partially be explained by microhomology (Fig. 2D). For the HBV integration hotspot at chr8, *EXT1* showed significantly higher expression in tumor tissue than in adjacent non-neoplastic liver tissues (Additional file 30: Fig. S15). In addition to stimulate HCC cell growth [42], higher expression of *EXT1* was associated with poor prognosis in lung, thyroid, and cervical cancers in TCGA. Together, these results suggest that the hotspots chr1: 34,397,059 (*CSMD2*) and chr8:118,557,327 (*MED30/EXT1*) were likely due to proliferation advantage of cells with these integrations over other cells. *EXT1* has been evaluated as a potential target in breast cancer [83] and multiple myeloma [84, 85]. Our results here support *EXT1* as a potential target in HBV-HCC. Further research is warranted to evaluate targeting *EXT1* in these types of cancers.

Our procedure for HBV integration site identification (“Methods” and Additional file 1: Methods) is based a Bayesian model with parameters tuned (Additional file 31: Fig. S16) to identify recurrent and sporadic integrations across single cells. In addition, CNVs are inferred from HBV genome-enriched single cell sequencing data. Both CNV analysis and cell evolution analysis suggest that 1q amplification, one of recurrent alterations in HCC [67], is a potential driver alteration (Figs. 3A and 4A) for this patient. The present results may have a profound impact on developing personalized treatment regimens for HBV-HCC. In this specific case, targeting EXT1, which is a driver of clonal expansion, means that some but not all clones may be killed. On the other hand, targeting 1q amplification, which is a putative root driver alteration, may lead to more tumor cells being killed. Thus, it is critical to distinguish between root driver alterations and ones for clonal expansion when developing precision drug treatments.

It is worth noting that the chimera read frequency in the HGE-scSeq dataset was 0.025%, which was much lower than the 6.19% reported by Tu et al*.* [47] and the 2–3% by Huang et al*.* [26] for MDA. Chimeras result from alternative secondary structures [86]. It is not clear whether the low chimera read frequency resulted from the HBV genome enrichment process [14]. Nevertheless, the number of chimera reads did not correlate with the number of reads on HBV or soft clipped reads, nor did it correlate with the number of reads on the human genome (Additional file 18: Fig. S3), suggesting that chimera reads had no impact on the HBV integration detection and copy number variation detection.

There are multiple limitations of the HGE-scSeq approach. Even though multiple data adjustment procedures were applied to make HBV integration detection procedure (Additional file 1: Methods, Additional file 31: Fig. S16) and CNV estimation procedure (Additional file 1: Methods, Additional file 32: Fig. S17, Additional file 33: Fig. S18, Additional file 34: Fig. S19) robust, the sensitivity of the approach is hard to estimate unless an extensive single cell whole genome sequencing is performed as the ground truth for comparison, which is expensive to do. Given the uncertainty of the sensitivity, it is not clear whether some tumor cells lacking chr1 or chr8 hotspot integrations were due to capture/sequencing sensitivity or due to clonal expansion. We compared two scenarios: (1) the root clone had HBV integration, which drives tumorigenesis. In this scenario, all clones should have the exact same HBV integration pattern (as HBV integration occurs only in the early phase of HBV integration [3, 4]), which contradicts with our observation that some clones had more HBV integrations than others (Fig. 3B). (2) the root clone had 1q amplification, and the root clone cells were of genome instability. Then, HBV infection occurred and HBV integration in each cell occurred at different sites and at different frequencies depending on each cell’s molecular state and genome stability, which is consistent with our observation (Fig. 3B). The cells in clone 4 were more likely to be missing the hotspot integrations than the cells in clone 1, suggesting that the lack of hotspot integration in these cells was unlikely to be due to the sensitivity of the assay, but rather to molecular differences between the clones. Similarly, the HBV integration site variations observed in MHCC97H cells could be due to errors introduced during genome multiplication and sequencing or due to true heterogeneity of cells in a cell line. Multiple HBV integrations were identified in more than one cell (Additional file 10: Table S9), suggesting that these HBV integrations were unlikely to have resulted from random sequencing errors. As HBV integrations only occur in early phase of HBV infection [3, 4] and are unlikely to be introduced after establishment of the MHCC97H cell line, the heterogeneity of HBV integration sites among individual cells suggests that the rare integrations may not have any impact on cell proliferation such that the composition of cells with different HBV integrations was stable during cell passage.

The relationship between CNVs and HBV integrations observed in this case study needs to be considered as anecdotal until the relationship can be replicated in more patient samples or validated in in vitro experiments that exceed the scope of this study.

## Conclusion

We developed a data analysis pipeline for HBV genome-enriched single cell sequencing data. HCC tumor cells were heterogeneous in terms of both HBV integration sites and CNVs. The frequency of HBV integration observed in the study was much higher than expected. For the HBV-HCC case in the study, multifocal tumors and tumor thrombi shared common HBV and CNV patterns, suggesting that they shared the same tumor origin.

## Supplementary Information

**Additional file 1: Methods.** Supplementary materials including supplementary methods.

**Additional file 2: Table S1.** Clinicopathological information of the patient. HCC, hepatocellular carcinoma; HBsAg, hepatitis B virus surface antigen; HBsAb, hepatitis B virus surface antibody; HBcAb, hepatitis B core antibody; HBeAb, hepatitis B e antibody; HCV Ab, hepatitis C virus antibody; AFP, alpha-fetoprotein; PVTT, portal vein tumor thrombosis; IVCTT, inferior vena cava tumor thrombosis. Hepatitis serology testing showed that the patient was HBsAb positive, HBsAg negative, HBcAb positive, HBeAb positive, HCV Ab negative and had no detectable blood HBV DNA copy number.

**Additional file 3: Table S2.** Reads distribution for HGE-scSeq from tumor, bulk tissue data from adjacent normal and HGE-scSeq from MHCC97H cells including number of raw reads, number of reads after filtering, number of reads pair-ended mapped to human genome, number of reads pair-ended mapped to HBV genome and number of soft clipped reads covering HBV integration site.

**Additional file 4: Table S3.** Coverage and width information for reads pair-ended mapped to human genome for HGE-scSeq from tumor, bulk tissue data from adjacent normal and HE-scSeq from from MHCC97H cells.

**Additional file 5: Table S4.** Match with Poisson distribution. Reads distribution on human genome is tested against Poisson distribution. The null hypothesis is the reads mapped to human genome following Poisson distribution. The test consistently fails until the corresponding region covering 88% of human genome.

**Additional file 6: Table S5.** Detected HBV virus and corresponding number of cells. The number of singles for each detected HBV sub strain is collected. The top 3 major HBV sub strains are HBV G247-B3 (GI121485896; GeneBank:EF134945.1), HBV strain Whutj-37 (GI38147024; GeneBank:AY293309.1) and HBV isolate G247-B5(GI:121485902; GeneBank:EF134946.1).

**Additional file 7: Table S6.** Pairwise alignment result with blat for the reference of top 5 most enriched HBV sub strain.

**Additional file 8: Table S7.** Correlation of CNVs detected with HGE-scSeq, WGS and SNP array based on Pearson, Spearman and Cosine.

**Additional file 9: Table S8.** HBV integrations detected with WGS from MHCC97H. At least one soft clipped read and two adjacent reads are required to call HBV integration.

**Additional file 10: Table S9.** HBV integrations detected with the HGE-scSeq of 5 MHCC97H cells. Highlighted integrations are matched with HBV integrations detected with WGS in 5000 bp range.

**Additional file 11: Table S10.** All detected Integration Events. All the detected HBV integration events for single cells from tumor and bulk tissue from adjacent normal. There are totally 471 HBV integrations observed from single cells in tumor and 17 HBV integrations observed from adjacent normal.

**Additional file 12: Table S11.** All unique integration sites. HBV integrations are merged if their position is within 20 bp. They are totally 164 unique HBV integrations for single cells from tumor and 13 unique HBV integrations for bulk tissue from normal. The gene annotation is provided by running ANNOVAR.

**Additional file 13: Table S12.** Integration hot spots supported by known fusion events. Hot spot genes are reported as cancer fusion gene by both cancer cell line and TCGA for different kinds of cancers.

**Additional file 14: Table S13.** Genome regions where CNV amplification is significantly associated with decreasing rate of rare HBV integration carrying cells, when focusing on chr11, whose CNV differentiated clone 1 vs. clones 2, 3, 4.

**Additional file 15: Table S14.** Genome regions where CNV amplification is significantly associated with decreasing rate of rare HBV integration carrying cells, when focusing on chr8:118268310–146364022, whose CNV differentiated clone 2 vs. clones 3, 4.

**Additional file 16: Fig. S1.** The location of tumors and thrombi on liver. **A** Magnetic resonance imaging (MRI) shows a 15 cm × 10 cm larger lesion in the left hepatic lobe and multiple smaller lesions in the right hepatic lobe, all less than 3 cm in diameter. Yellow arrows indicate multiple tumor foci of various sizes. **B** MRI with contrast enhancement reveals tumor thrombosis involving the inferior vena cava (IVCTT), and the right portal vein branch (PVTT), indicated by the red arrows, respectively, suggesting intrahepatic and extrahepatic vascular spread of HCC.

**Additional file 17: Fig. S2.** Data analysis flow chat. **A** General analysis flow chat. After filtering low quality raw reads and detecting the HBV sub strain. HBV integrations and single cell CNV are called separately. **B** Pipeline for detecting HBV integration. **C** Pipeline for detecting single cell CNV.

**Additional file 18: Fig. S3.** Histograms of number of human reads (**A**), number of HBV reads (**B**), number of inter chromosome chimera reads (**C**), number of intra chromosome chimera reads (**D**), number of softclipped reads (**E**). The average chimera reads ratio is 0.025% which is lower than the reported chimera reads ratio of 6.19% by Tu et.al and 2%/3% by Xie’s group. **F** Correlation coefficients between the numbers of human reads, inter chromosome chimera reads, intra chromosome chimera reads, HBV integrations, and HBV reads. Numbers of chimera reads for inter and intra chromosome are highly correlated. Numbers of chimera reads are not correlated with number of reads on HBV, number of soft clipped reads and number of reads on human. Numbers of reads on HBV and soft clipped reads are correlated.

**Additional file 19: Fig. S4.** Distribution of number of cells with reads covering the each loci. Red line indicates the mean. Each bin corresponds to the fraction of human genome is successfully sequenced in a number of cells. If the reads distribute randomly on human genome, the distribution follows Poisson distribution.

**Additional file 20: Fig. S5.** **A** Compare HBV sequence and human genome sequence with Fisher values. **B** Fisher values from Human mapped region. **C** Fisher Values from Human unmapped region.

**Additional file 21: Fig. S6.** MHCC97H’s CNV profile generated by enriched single cell sequencing, whole genome sequencing and SNParray.

**Additional file 22: Fig. S7.** Distribution of number of cells with reads covering the each loci for MHCC97H. Each bin corresponds to the fraction of human genome is successfully sequenced in a number of cells. If the reads distribute randomly on human genome, the distribution follows Poisson distribution. Chi-square test against Poisson distribution producing p-value 0.98.

**Additional file 23: Fig. S8.** Distribution of repeatedly covered loci across the copy number amplified region called from Whole genome sequence data for MHCC97H.

**Additional file 24: Fig. S9.** **A** Compare HBV sequence and human genome sequence with Fisher values. **B** Fisher values from Human mapped region for MHCC97H. **C** Fisher Values from Human unmapped region for MHCC97H.

**Additional file 25: Fig. S10.** **A** HBV reads pileup results for an example cell with IGV. The reference genome is G247-B3. HBx-protein region is labeled as red. **B** HBV reads pileup results comparing between tumor tissues and adjacent normal tissues. The upper panel is for all the HBV reads in adjacent normal tissues. The lower panel is for all the HBV reads in tumor tissues.

**Additional file 26: Fig. S11.** Linear correlation between inter chromosome chimera reads, intra chromosome chimera reads and length of chromosomes. Scatter plots (**A**, **C**) and boxplot (**B**, **D**) of number of chimera reads and length of chromosome for both inter and intra chromosome cases. The blue triangles indicate Chr1 and Chr8. The numbers in A and C are (correlation between chromosomes’ length and mean # of chimera reads | p-value) and (correlation between chromosomes’ length and median # of chimera reads | p-value). The correlations between numbers of chimera reads and length of chromosome are significant.

**Additional file 27: Fig. S12.** **A** Distribution of HBV integrations across HBV proteins of P, S, X, C. HBV integrations are located on S, C and X. **B** Distribution of HBV integrations across HBV genome.

**Additional file 28: Fig. S13.** Compare the read throughput of the two clustered sets of cells from Fig. 2C. Histograms of reads throughput from these two sets of cells are almost overlapped. K.S. test shows no significant difference between these two distributions. The set of cells carrying extra integrations other than hot spot integrations are not benefit from higher throughput of reads.

**Additional file 29: Fig. S14.** Labeling the phylogenetic tree in Fig. 4A by carrying only hot spot integrations, extra rare integrations and no integrations. We can find that with dynamic clonal evolution. The rate of rare integration is becoming less and less.

**Additional file 30: Fig. S15.** Expression of the hot spot genes from ICGC and TCGA. Hot spot genes *CSMD2, MED30*, and *EXT1* are find expressed significantly higher in tumor samples then adjacent normal samples.

**Additional file 31: Fig. S16.** **A** Find the best tuning parameter for the pseudo count and weight adjustment. **B** Select the best cutoff for the selected best tuning parameter.

**Additional file 32: Fig. S17.** Quality of Bin's read count correction. **A** Fold enrichment of top x% bins carrying HBV integration before correction. Bins are sorted by the number of reads mapped in the bin. **B** Fold enrichment of top % bins carrying HBV integration after correction. Bins are sorted by corrected reads. **C** MAPD and MAD before batch effect correction. **D** MAPD and MAD after batch effect correction.

**Additional file 33: Fig. S18.** Comparison of the number reads between normal bulk tissues and tumor single cells. Histogram shows the distribution for tumor single cells while vertical color lines show the corresponding quantity of normal control tissue. **A** Comparison of numbers of filtered reads; **B** comparison of percentage of reads mapped to human genome; **C** comparison of coverage on human genome; **D** comparison of width on human genome.

**Additional file 34: Fig. S19.** **A** Comparison dispersion of binned reads count after mappability and GC content correction between the smallest one in single tumor cells and the four normal control tissue. **B** CNV results on normal tissues.

## Data Availability

The datasets generated and/or analyzed during the current study are available in the NIH SRA (BioProject: PRJNA553308). The human genome assemble hg19 used in the study is downloaded from https://hgdownload.soe.ucsc.edu/goldenPath/hg19/chromosomes/. The collection of 32,102 virus genomes of all classes used in the study is a part of the RINS (https://s3.amazonaws.com/changseq/kqu/rins/rins.tar.gz) package [52].

## Abbreviations

HBV: Hepatitis B Virus
HCC: Hepatocellular carcinoma
WGS: Whole genome sequencing
HIVID: High-throughput Viral Integration Detection
HPV: Human papillomavirus
CNVs: Copy number variations
MALBAC: Multiple annealing and looping-based amplification cycles
MDA: Multiple displacement amplification
WGA: Whole Genome Amplification
HGE-scSeq: HBV genome-enriched Single cell sequencing
