# Supplementary material for: HBV genome-enriched single cell sequencing revealed heterogeneity in HBV-driven hepatocellular carcinoma (HCC)
Source: BMC Med Genomics. 2022 Jun 16;15:134. doi: 10.1186/s12920-022-01264-2 (PMC9205089; doi:10.1186/s12920-022-01264-2)
Supplement: Supplementary file 1 — Additional file 1: Methods. Supplementary materials including supplementary methods. [file 12920_2022_1264_MOESM1_ESM.docx]

**Supplementary Materials for**

**HBV genome-enriched single cell sequencing revealed heterogeneity in HBV-driven hepatocellular carcinoma (HCC)**

Wenhui Wang^1,2,3†^, Yan Chen^4†^, Liang Wu^5^, Yi Zhang^6^, Seungyeul Yoo^1,2,3^, Quan Chen^1,2,3^, Shiping Liu^5^, Yong Hou^5^, Xiao-ping Chen^4^, Qian Chen^7*^, Jun Zhu^1,2,3,8*^,

^1^Department of Genetics and Genomic Sciences, Icahn School of Medicine at Mount Sinai, New York, NY, United States

^2^Icahn Institute for Genomics and Multiscale Biology, Icahn School of Medicine at Mount Sinai, New York, NY, United States

^3^Sema4, Stamford, Connecticut, United States

^4^The Hepatic Surgery Centre at Tongji Hospital, Tongji Medical College, Huazhong University of Science and Technology (HUST), Wuhan, China

^5^BGI, Shenzhen, China

^6^Department of Mathematics, Hebei University of Science and Technology, Shijiazhong, Hebei, China

^7^The Division of Gastroenterology, Department of Internal Medicine at Tongji Hospital, Tongji Medical College, Huazhong University of Science and Technology (HUST), Wuhan, China.

^8^The Tisch Cancer Institute, Icahn School of Medicine at Mount Sinai, New York, NY, United States

^†^co-first authors with equal contribution

^*^Corresponding Authors:

Dr. Jun Zhu

Department of Genetics and Genomic Sciences,

Icahn School of Medicine at Mount Sinai,

1425 Madison Ave. New York, NY, United States. 10029

Tel: 212-659-8942

Email: jun.zhu@mssm.edu

Dr. Qian Chen

The Division of Gastroenterology, Department of Internal Medicine at Tongji Hospital,

Tongji Medical College, Huazhong University of Science and Technology (HUST),

Wuhan, China

Email: [chenqian201579@yahoo.com](mailto:chenqian201579@yahoo.com)

**Supplementary Methods**

***Detecting HBV integration sites***

Reads not mapped to the human genome and virus genome were assembled to long reads and were aligned to the human genome and virus genome, then the soft clipped reads were collected for identifying the integration sites as described in Methods. As the number of soft clipped reads and the number of HBV reads were correlated (Supplementary Figure S3F). We normalized soft clipped read against the number of HBV reads. The Optimal threshold of soft clipped reads for HBV integration was selected to minimize the correlation between numbers of HBV reads and detected HBV integrations. Further refinement based on Bayesian model was used to identify recurrent HBV integrations. Steps are detailed as following:

1. We collect soft clipped reads into a matrix $A$ of$m\times n$, *m* is the number of cells, *n* is the number of candidate integration sites. $A(i,j)$is the number of soft clipped reads for cell *i* on site *j*. Only cell *i* and site *j* was included if $\sum_{i=1}^{m} A(i,j)>0$ and $\sum_{j=1}^{n} A(i,j)>0$. As result, *n*=1108 sites and *m*=189 cells were included for our data.
2. We collect the number of HBV reads $H_{m\times1}$ in each cell. $H_{i}$is the number of HBV reads in cell *i*.
3. We normalize the number of soft clipped reads against the number of HBV reads as

$A_{i,j}^{1}=\frac{A_{i,j}}{(H_{i}\times100)/\mathrm{L}_{\mathrm{HBV}}}10000$, where the denominator is the estimation of HBV load. The number of soft clipped reads is normalized with the same load of HBV, or the same scale of HBV probe enrichment.

1. We rank order $A^{1}[A\geq2]$and convert them into 1 to 100 quantiles as the probability of integration as$P_{i,j}^{1}$ . At this step, we only consider integrations supported with at least 2 soft clipped reads in a cell.
2. Next, we search for the optimal cutoff value at which the total number of integration sites is least depended on the total number of HBV reads. For each quantile cutoff *k*, calculate the correlation coefficient between the number of integrations and number of HBV reads as the following:

$r(k)=corr(\sum_{j} (P_{i,j}^{1}>k/100),H_{i})$ .

At the cutoff $k$ corresponding to lowest absolute correlation coefficient, we identified 141 cells carrying HBV integrations on 164 unique sites, which are denoted as *INT_SITE1*. Among them, 31 integrations were identified in more than one cell. There are totally 427 HBV integration events, which are denoted as *INT_EVENT1*.

1. Then, we tried to identify more recurrent integrations. We implemented a Pseudo Count Weight Adjustment (PCWA) to rescue the potential false negative integrations given supporting reads in other cells. The pseudo count weight matrix $W_{m\times n}$is defined as: $W_{i,j}=sqrt(\sum_{i\neq j,i\leq m} A_{i,j}^{1})$.

The pseudo count weight adjusted score $S_{m\times n}$is defined as: $S_{i,j}=1-e^{-A_{i,j}^{1}(1+\alpha W_{i,j})}$. $\alpha$is the tuning parameter used to adjust the contribution from pseudo count weight.

1. For a given$\alpha$, a score matrix $S_{m\times n}(\alpha)$is generated. Following step 5, we convert $S_{m\times n}(\alpha)$into 1 to 100 quantiles as the probability of integration as$P_{i,j}^{2}(\alpha)$. A cutoff is needed to call integrations.
2. In order to find the optimal cutoff *k* which recovers most repeated integrations while incorporates random integrations and misses detected integrations as few as possible, we select the following optimization criteria:

$$val_{\alpha}(k)=\frac{N\_gain(k)}{(N\_gain\_random(k)+1)\times(N\_loss(k)+1)}$$

, where

$N\_gain_{\alpha}(k)=\#\{P_{i,j}^{2}(\alpha)>k/100,j\in INT\_STIE1\}\cap\overline{INT\_EVENT1}$ is the number of new integrations above threshold at the sties included in the first round;

$N\_gain\_random_{\alpha}(k)=\#\{P_{i,j}^{2}(\alpha)>k/100,j\notin INT\_STIE1\}\cap\overline{INT\_EVENT1}$is the number of new integrations above threshold at the sites NOT included in the first round;

$N\_loss_{\alpha}(k)=\#\{P_{i,j}^{2}(\alpha)<k/100\}\cap INT\_EVENT1$is the number of integrations below threshold for integrations included in the first round. And k is selected as the cutoff producing the maximum validation score $val_{\alpha}(k)$ as $k=\mathop{\mathrm{argmax}}\limits_{1\leq k\leq100} \{val_{\alpha}(k)\}$

1. For a given$\alpha$, the maximum validation score$\max_{1\leq k\leq100} \{val_{\alpha}(k)\}$indicates the best performance with the$\alpha$. Therefore, we define the validation score for $\alpha$as $VAL(\alpha)=\max_{1\leq k\leq100} \{val_{\alpha}(k)\}$.
2. We searched $\alpha$on$[10^{-6},2\times10^{-6}...10^{-4},2\times10^{-4},...,0.01,0.02,...1,2...100]$. We find that the best alpha arrive at 0.26 (Supplementary Figure S16A). Corresponding to the best cutoff, N_gain=44, N_gain_random=4, N_loss=1 (Supplementary Figure S16B).
3. The 44 new recovered HBV integration events are merged to the results from step 5. Finally, HBV integrations are detected on 164 unique sites and 142 cells. 39 of HBV integrations are repeatedly discovered.

***Estimating copy number variations***

Reads mapped to human genome were randomly distributed. To consider biases in the sequencing data w based on an enriched single cell sequencing protocol [[1](#_ENREF_1)], a pipeline for inferring copy number variations is developed by adapting the method reported by Baslan *et al* [[2](#_ENREF_2)].

Kuilman *et al.* [[3](#_ENREF_3)] shows that off-target reads from enriched sequencing can be used to obtain DNA copy number profiles by removing peaks of mapped reads and compensating according to the size of peaks and average local coverage. However, Kuilman *et al*.’s method [[3](#_ENREF_3)] was not directly applicable for this data set due to the sparsity of reads covered region originated from single cell whole genome amplification. Baslan *et al.* [[2](#_ENREF_2)] describes a procedure characterizing single cell copy number variation based on flow sorting of single nuclei, whole genome amplification and next generation sequencing. An informatics workflow of inferring CNV from the raw single cell sequencing data is outlined in Supplementary Figure S2C. In addition to correction for mappability, removal of duplication, and GC content normalization, we used soap [[4](#_ENREF_4)] as the alignment software to be consistent with the alignment software used in HIVID.

There are following steps in the pipeline for calling CNV:

1). The sequence of pseudo autosomal regions on chrY was changed to N. The sequence of pseudo autosomal regions on chrY is exactly the same as the corresponding regions on chrX. Generate the index of the reference genome for soap with 2bwt-builder.

2). Sequencing reads were simulated based on the reference genome. Starting from the first position of a chromosome a fragment of 100 bases was extracted to generate a sequencing read. The step continued at the following positions until the end of the chromosome.

3). The simulated sequencing reads were mapped to the modified human reference genome with soap (-s 85 -l 50 -v 2 -r 1 -p 6 -m 100 -x 500)

4) Genome positions with simulated sequencing reads mapped uniquely back to where they were extracted from were defined as mappable positions. All the mappable positions were collected, and the number of mappable positions on each chromosome was counted.

5) Mappable positions were grouped into 5000 bins. Number of bins allocated to each chromosome was proportional to the number of mappable positions on that chromosome. The number of mappable positions for each bin was computed by dividing the number of mappable positions on the chromosome with the number of allocated bins on that chromosome. The boundaries of bins were decided by the number of mappable positions in each bin sequentially on the chromosome. The average length of bin is 560485.9 (sd: 989.8). If more than 50% of a bin overlaps with bad bins reported in the paper [[2](#_ENREF_2)], the bin is defined as a bad bin. These bad bins are mainly located at centromere regions of chromosomes. As a result, 11 bad bins were filtered out.

6) The GC content in each bin was calculated.

7) The filtered reads were mapped to reference human genome with soap (-s 85 -l 50 -v 2 -r 1 -p 6 -m 100 -x 500)

8) The result of pair end aligned reads was converted to sam format with soap2sam.pl, then to bam format with samtools. The duplicated reads were removed with Picard.

9) The number of reads in each bin was counted and normalized by dividing the mean read count of a cell.

10) GC content was normalized using LOWESS smoothing. In brief, a regression model was constructed by regressing the read count against the GC content percentage with LOWESS regression. Then, the corrected read count is calculated by minus the input read count with the one predicted by the regression model on the corresponding GC percentage.

11) The read counts after mappability and GC content normalization were collected into a 269 by 4989 matrix M, where 269 was the number of cells and 4989 was the number of bins. The index of dispersion for each cell was calculated as the ratio between standard deviation and mean of bin’s read count. As suggested by Garvin *et al.* [[5](#_ENREF_5)], the sample with lowest index of dispersion is mostly likely to be the cell with the most balanced ploidy. Therefore, the read count profiles of all cells were normalized against the one having lowest index of dispersion with LOWESS.

12) Similar to Gao *et al.* [[6](#_ENREF_6)], outliers were removed with R function *winsorize*. Then, a multiple sample population segmentation algorithm with default parameter [[7](#_ENREF_7)] was used to call the segments under the condition that these cells are related. Segments with less than 10 bins were removed and the neighbor segments were joined or separated in the middle of removed segment if they differed significantly. At the end, bins were merged into a total of 49 segments.

13) A least-square rounding method was used to get the optimum scaling factor that had the least sum of deviations from the closet integer after rounding. Integer copy number status was further classified into 3 cases of loss, normal and amplification and denoted as -1, 0, 1.

***Evaluation of read count correction***

Because sequences containing HBV sequence were enriched at the DNA library preparation step, we need to correct read count bias due to enrichment sequencing. For each cell, we collected the rank of read counts for bins with HBV integrations detected. Then, for all the bins with HBV integrations, we calculated the fraction of bins with rank higher than$\alpha\%$,$f(\alpha)$, where $\alpha$is ranging from 1 to 100. Then the fold enrichment of top $\alpha$% ranked bins among bins with HBV integration is defined as$100\times f(\alpha)/\alpha$. In an ideal case where there is no bias from enrichment sequencing, the fold enrichment should be around 1.

Two metrics to characterize the overall quality of binned reads were introduced. Assuming there are *n* bins, the number of reads in each bin is *count*[*k*] (*k*=1..*n*). The average number of reads across bins is *C*. Garvin et. al [[5](#_ENREF_5)] introduce median absolution deviation (MAD) to quantify the uniformity of bin’s read count. For each cell, MAD is defined as$MAD=median\{|\frac{count[k]}{C}-\frac{count[k-1]}{C}|, k=2...n\}$. MAD is expected to reflect the bin count dispersion due to technical noise. Another metric named as MAPD[[8](#_ENREF_8)] is defined as $MPAD=median\{|\log_{2}(\frac{count[k]}{C})-\log_{2}(\frac{count[k-1]}{C})|, k=2...n\}$, which is originally used as a QC metric for microarray data. Cai et al. [[9](#_ENREF_9)] propose to utilize MAPD [[8](#_ENREF_8)] to measure the quality of read counts in bins. MAPD is shown to be more robust to identify true CNVs [[9](#_ENREF_9)]. An optimal threshold of 0.45 is suggested by Cai et al. [[9](#_ENREF_9)], which is also used in other studies [[10](#_ENREF_10)].

Supplementary Figure S17A&B are the bar plots of fold enrichment of top ɑ% ranked bins according to reads count across all the bins with HBV integration. The bins with HBV integration were enriched for top ranked bins according to raw read count (Supplementary Figure S17A). The highest fold enrichment was for top 1% ranked bins, which was as high as 19.48. The read counts after normalization are showed in Supplementary Figure S17B. The highest fold enrichment was 2.03, which means the bias of reads count from enrichment sequencing had been successfully corrected. The Supplementary Figure S17C &D show the box plot of MAD and MAPD. The Supplementary Figure S17C shows MAPD and MAD for the reads count after mappabiliy and GC content correction, and Supplementary Figure S17D shows the read count after further corrected by the reads count from the cell with least dispersion. Both MAD and MAPD were significantly decreased after the corrections. At the threshold of 0.45 suggested by Cai *et al.* [[9](#_ENREF_9)] for filtering the cells with low reads quality, only 3 cells passed the threshold before the least dispersion sample correction, while all the cells passed the threshold after the least dispersion sample correction.

***Evaluating the CNV pipeline with reads from normal control***

As our CNV pipeline was modified from a CNV pipeline for single cell sequencing data, which takes full consideration of correcting for bias incorporated from WGA [[11](#_ENREF_11)]. Whether our modified pipeline can handle bulk tissue enrichment sequencing data needs to be evaluated.

As shown above, the reads mapped to human genome for normal control tissue resulted in higher average coverage and width comparing to ones for tumor single cells. However, the improvement on coverage and width was not large. The cases for normal control tissue and tumor single cell were comparable (Supplementary Figure S18). Thus, the sequencing data for normal tissue samples can be used for evaluating the performance of our CNV pipeline in correcting the bias generated from enriched sequencing.

The dispersion of the binned reads counts for the four adjacent normal liver tissue samples after mappability and GC content correction was lower than the smallest corresponding dispersion in tumor single cells (Supplementary Figure S19A). Therefore, mappability correction and GC content correction for the normal control tissue data were necessary. Most of the regions across the 4 adjacent normal tissue samples were of normal copy number (Supplementary Figure S19B). Therefore, our CNV pipeline for correcting the potential bias due to enriched sequencing step was validated.

***Association between clone evolution and HBV integrations***

Parsimony method is mostly recommended for constructing phylogenetic trees from single cell CNV profiles [[6](#_ENREF_6), [12](#_ENREF_12)]. The distance based tree building method generally assumes that evolution drives by mutations independently accumulated one at a time. CNVs estimated in our study contain multiple alterations. Therefore, in this study, we used a parsimony method [[6](#_ENREF_6)] to build phylogenetic trees based on CNVs at the 49 identified CNV segments.

We identified 4 putative clones according to copy number profiles (Figure 3A). Meanwhile, there were two categories for cells based on HBV integration, cells with only hot spot integrations and cells with extra rare integrations. There was a clear trend that the ratio of cells carrying rare integrations decreased when number of regions with DNA copy number amplification increased (Figure 3B).

Putative clones are identified according to CNV and HBV integration profiles. A phylogenetic tree based test of association method was used to decode the association between specific CNV and ratio for cells with rare HBV integrations. First, inner node in the phylogenetic tree that separate parent and child clones which are identified by hierarchical clustering was detected. Second, copy number variations at the genomic region corresponding to the detected inner node for the cells belonging to parent and child clones were collected. Third, a contingency table was built based on numbers of cells with extra rare HBV integrations or only HBV integrations at hot spots, copy number amplification, copy number normal. Fourthly, Fisher’s exact test p-value corrected by multiple testing was used to assess the association. Last, functional enrichment analysis by DAVID [[13](#_ENREF_13)] was used to annotate genes in the CNV bins that were significantly associated with rare HBV integration events.

For example, CNVs on Chr11 which differentiated Clone 1 and Clones 2-4 were identified based on the phylogenetic tree shown in Figure 4A. Next, the cells from related clones (Clone 1 and Clones 2-4) were compared at bins within the genomic signaling region. For each bin in the region, a contingency table was built to test the association between hot spot integration vs. rare integration and copy number amplification vs. normal. Bins of FDR<0.05 were collected and annotated for potential enriched functions. Last, genes located on the significant regions were used as input for functional enrichment analysis.

**References**

1. Li W, Zeng X, Lee NP, Liu X, Chen S, Guo B, Yi S, Zhuang X, Chen F, Wang G *et al*: **HIVID: an efficient method to detect HBV integration using low coverage sequencing**. *Genomics* 2013, **102**(4):338-344.

2. Baslan T, Kendall J, Rodgers L, Cox H, Riggs M, Stepansky A, Troge J, Ravi K, Esposito D, Lakshmi B *et al*: **Genome-wide copy number analysis of single cells**. *Nat Protoc* 2012, **7**(6):1024-1041.

3. Kuilman T, Velds A, Kemper K, Ranzani M, Bombardelli L, Hoogstraat M, Nevedomskaya E, Xu G, de Ruiter J, Lolkema MP *et al*: **CopywriteR: DNA copy number detection from off-target sequence data**. *Genome biology* 2015, **16**:49.

4. Li R, Yu C, Li Y, Lam T-W, Yiu S-M, Kristiansen K, Wang J: **SOAP2: an improved ultrafast tool for short read alignment**. *Bioinformatics* 2009, **25**(15):1966-1967.

5. Garvin T, Aboukhalil R, Kendall J, Baslan T, Atwal GS, Hicks J, Wigler M, Schatz MC: **Interactive analysis and assessment of single-cell copy-number variations**. *Nature methods* 2015, **12**(11):1058-1060.

6. Gao R, Davis A, McDonald TO, Sei E, Shi X, Wang Y, Tsai PC, Casasent A, Waters J, Zhang H *et al*: **Punctuated copy number evolution and clonal stasis in triple-negative breast cancer**. *Nature genetics* 2016, **48**(10):1119-1130.

7. Nilsen G, Liestol K, Van Loo P, Moen Vollan HK, Eide MB, Rueda OM, Chin SF, Russell R, Baumbusch LO, Caldas C *et al*: **Copynumber: Efficient algorithms for single- and multi-track copy number segmentation**. *BMC genomics* 2012, **13**:591.

8. Affymetrix: **Median of the absolute values of all pairwise differences and quality control on Affymetrix genome-wide human SNP array 6.0.** In*.*; 2008.

9. Cai X, Evrony GD, Lehmann HS, Elhosary PC, Mehta BK, Poduri A, Walsh CA: **Single-cell, genome-wide sequencing identifies clonal somatic copy-number variation in the human brain**. *Cell Rep* 2014, **8**(5):1280-1289.

10. Ning L, Li Z, Wang G, Hu W, Hou Q, Tong Y, Zhang M, Chen Y, Qin L, Chen X *et al*: **Quantitative assessment of single-cell whole genome amplification methods for detecting copy number variation using hippocampal neurons**. *Scientific reports* 2015, **5**:11415.

11. Gawad C, Koh W, Quake SR: **Single-cell genome sequencing: current state of the science**. *Nature reviews Genetics* 2016, **17**(3):175-188.

12. Schwartz R, Schaffer AA: **The evolution of tumour phylogenetics: principles and practice**. *Nature reviews Genetics* 2017, **18**(4):213-229.

13. Huang DW, Sherman BT, Lempicki RA: **Systematic and integrative analysis of large gene lists using DAVID bioinformatics resources**. *Nature protocols* 2009, **4**(1):44-57.
