# Supplementary material for: HBV genome-enriched single cell sequencing revealed heterogeneity in HBV-driven hepatocellular carcinoma (HCC)
Source: BMC Med Genomics. 2022 Jun 16;15:134. doi: 10.1186/s12920-022-01264-2 (PMC9205089; doi:10.1186/s12920-022-01264-2)
Supplement: Supplementary file 2 — Additional file 2: Table S1. Clinicopathological information of the patient. HCC, hepatocellular carcinoma; HBsAg, hepatitis B virus surface antigen; HBsAb, hepatitis B virus surface antibody; HBcAb, hepatitis B core antibody; HBeAb, hepatitis B e antibody; HCV Ab, hepatitis C virus antibody; AFP, alpha-fetoprotein; PVTT, portal vein tumor thrombosis; IVCTT, inferior vena cava tumor thrombosis. Hepatitis serology testing showed that the patient was HBsAb positive, HBsAg negative, HBcAb positive, HBeAb positive, HCV Ab negative and had no detectable blood HBV DNA copy number. [file 12920_2022_1264_MOESM2_ESM.docx]

**Supplementary Table S1**. Clinicopathological information of the patient. HCC, hepatocellular carcinoma; HBsAg, hepatitis B virus surface antigen; HBsAb, hepatitis B virus surface antibody; HBcAb, hepatitis B core antibody; HBeAb, hepatitis B e antibody; HCV Ab, hepatitis C virus antibody; AFP, alpha-fetoprotein; PVTT, portal vein tumor thrombosis; IVCTT, inferior vena cava tumor thrombosis. Hepatitis serology testing showed that the patient was HBsAb positive, HBsAg negative, HBcAb positive, HBeAb positive, HCV Ab negative and had no detectable blood HBV DNA copy number.

| Etiology |  |
| --- | --- |
| Alcohol Abuse/Drug Abuse | - |
| Mother-to-child Transmission of Hepatitis | - |
| Blood Transfusion | - |
| Virus Infection |  |
| HBsAg expression | - |
| HBsAb expression | - |
| HBcAb expression | + |
| HBeAb expression | + |
| HBV-DNA | Undetectable |
| HCV-Ab expression | - |
| Tumor marker, AFP (ng/mL) | 1210 |
| Pathological Features |  |
| No. of Tumors | 4 |
| Size of Largest Tumor, cm | 15cm x 10cm |
| Histologic Type of Tumors | Trabecular, Solid |
| Histology of Non-tumor Tissue | Cirrhosis |
| Tumor Grade | Moderately, Poorly |
| Intrahepatic Vessels Invasion | +, PVTT |
| Extrahepatic Vessels Invasion | +, IVCTT |
| TNM Stage | Stage IV |
