# Supplementary material for: HBV genome-enriched single cell sequencing revealed heterogeneity in HBV-driven hepatocellular carcinoma (HCC)
Source: BMC Med Genomics. 2022 Jun 16;15:134. doi: 10.1186/s12920-022-01264-2 (PMC9205089; doi:10.1186/s12920-022-01264-2)
Supplement: Supplementary file 6 — Additional file 6: Table S5. Detected HBV virus and corresponding number of cells. The number of singles for each detected HBV sub strain is collected. The top 3 major HBV sub strains are HBV G247-B3 (GI121485896; GeneBank:EF134945.1), HBV strain Whutj-37 (GI38147024; GeneBank:AY293309.1) and HBV isolate G247-B5(GI:121485902; GeneBank:EF134946.1). [file 12920_2022_1264_MOESM6_ESM.docx]

Supplementary Table S5. Detected HBV virus and corresponding number of cells. The number of singles for each detected HBV sub strain is collected. The top 3 major HBV sub strains are HBV G247-B3 (GI121485896; GeneBank:EF134945.1), HBV strain Whutj-37 (GI38147024; GeneBank:AY293309.1) and HBV isolate G247-B5(GI:121485902;GeneBank:EF134946.1).

| Virus | number of cells |
| --- | --- |
| HBV G247-B3 (GI121485896; GeneBank:EF134945.1) | 61 |
| HBV strain Whutj-37 (GI38147024; GeneBank:AY293309.1) | 34 |
| HBV isolate G247-B5(GI:121485902;GeneBank:EF134946.1) | 31 |
| HBV clone 712-64 (GI:164654564; GeneBank: EU306695.1) | 28 |
| HBV isolate D54 (GI:197292958; GeneBank:EU939676.1) | 14 |
| HBV isolate Ia-4 (GI:51449932; GeneBank:AY596104.1) | 7 |
| HBV isolate J180 (GI260184246; GeneBank:GQ377612.1) | 6 |
| HBV DNA strain: B0503341(PTK)F (GI:251822119; GeneBank;AP011084.1) | 4 |
| HBV clone 712-47 (GI:164654572; GeneBank: EU306696.1) | 3 |
| HBV isolate FH10 (GI:18252568; GeneBank:AF461362.1) | 2 |
| HBV isolate GZ-JSM (GI:90994710; GeneBankDQ448622.1) | 2 |
| HBV clone 712-99 (GI:164654580; GeneBankEU306697.1) | 2 |
| HBV isolate C150 (GI:197292916; GeneBankEU939666.1) | 1 |
| HBV isolate J81 (GI:260183955; GeneBank:GQ377550.1) | 1 |
| HBV isolate Bc (GI:241994936; GeneBank:GQ205440.1) | 1 |
| HBV isolate B1r-4 (GI:29124870; GeneBankAY217357.1) | 1 |
| HBV DNA isolate: HBV-IF687-1 (GI:60279587; GeneBankAB205119.1) | 1 |
| HBV clone 220-27 (GI:164654457; GeneBank:EU306680.1) | 1 |
| HBV isolate HBV B2(GI:9454475; GeneBank:AF282918.1) | 1 |
| HBV clone 807-13 (GI:164654595; GeneBank:EU306699.1) | 1 |
| HBV isolate J105 (GI:260184042; GeneBank:GQ377569.1) | 1 |
| HBV isolate Q6-5 (GI:29124880; GeneBank:AY217359.1) | 1 |
| HBV isolate C28 (GI:197292806; GeneBankEU939639.1) | 1 |
