# Supplementary material for: HBV genome-enriched single cell sequencing revealed heterogeneity in HBV-driven hepatocellular carcinoma (HCC)
Source: BMC Med Genomics. 2022 Jun 16;15:134. doi: 10.1186/s12920-022-01264-2 (PMC9205089; doi:10.1186/s12920-022-01264-2)
Supplement: Supplementary file 7 — Additional file 7: Table S6. Pairwise alignment result with blat for the reference of top 5 most enriched HBV sub strain. [file 12920_2022_1264_MOESM7_ESM.docx]

**Supplementary Table S6**. Pairwise alignment result with blat for the reference of top 5 most enriched HBV sub strain. Length of match/Length of HBV in each cell.

|  | **B3** | **B5** | **Whutj-37** | **712_64** | **D54** |
| --- | --- | --- | --- | --- | --- |
| B3 |  | 6/3215 | 38/3215 | 43/3215 | 54/3215 |
| B5 |  |  | 38/3215 | 43/3215 | 56/3215 |
| Whutj-37 |  |  |  | 40/3215 | 52/3215 |
| 712_64 |  |  |  |  | 56/3215 |
| D54 |  |  |  |  |  |
