# Supplementary material for: HBV genome-enriched single cell sequencing revealed heterogeneity in HBV-driven hepatocellular carcinoma (HCC)
Source: BMC Med Genomics. 2022 Jun 16;15:134. doi: 10.1186/s12920-022-01264-2 (PMC9205089; doi:10.1186/s12920-022-01264-2)
Supplement: Supplementary file 13 — Additional file 13: Table S12. Integration hot spots supported by known fusion events. Hot spot genes are reported as cancer fusion gene by both cancer cell line and TCGA for different kinds of cancers. [file 12920_2022_1264_MOESM13_ESM.docx]

Supplementary Table S12. Integration hot spots supported by known fusion events. Hot spots gene are reported as cancer fusion gene by both cancer cell line and TCGA for different kinds of cancers.

| **Hot Spot** | **Cell line fusion** | **TCGA** |
| --- | --- | --- |
| CSMD2 | G111 (Brain), YMB-1 (Breast) | LUAD, OV |
| MED30-EXT1 | EXT1: SCLC-2H (Lung); SNU-423 (Liver); HCC1395(Breast) | EXT1: BLCA;BRCA(2); OV UCEC; LGG |
