# Supplementary figures and images for: HBV genome-enriched single cell sequencing revealed heterogeneity in HBV-driven hepatocellular carcinoma (HCC)
Source: BMC Med Genomics. 2022 Jun 16;15:134. doi: 10.1186/s12920-022-01264-2 (PMC9205089; doi:10.1186/s12920-022-01264-2)

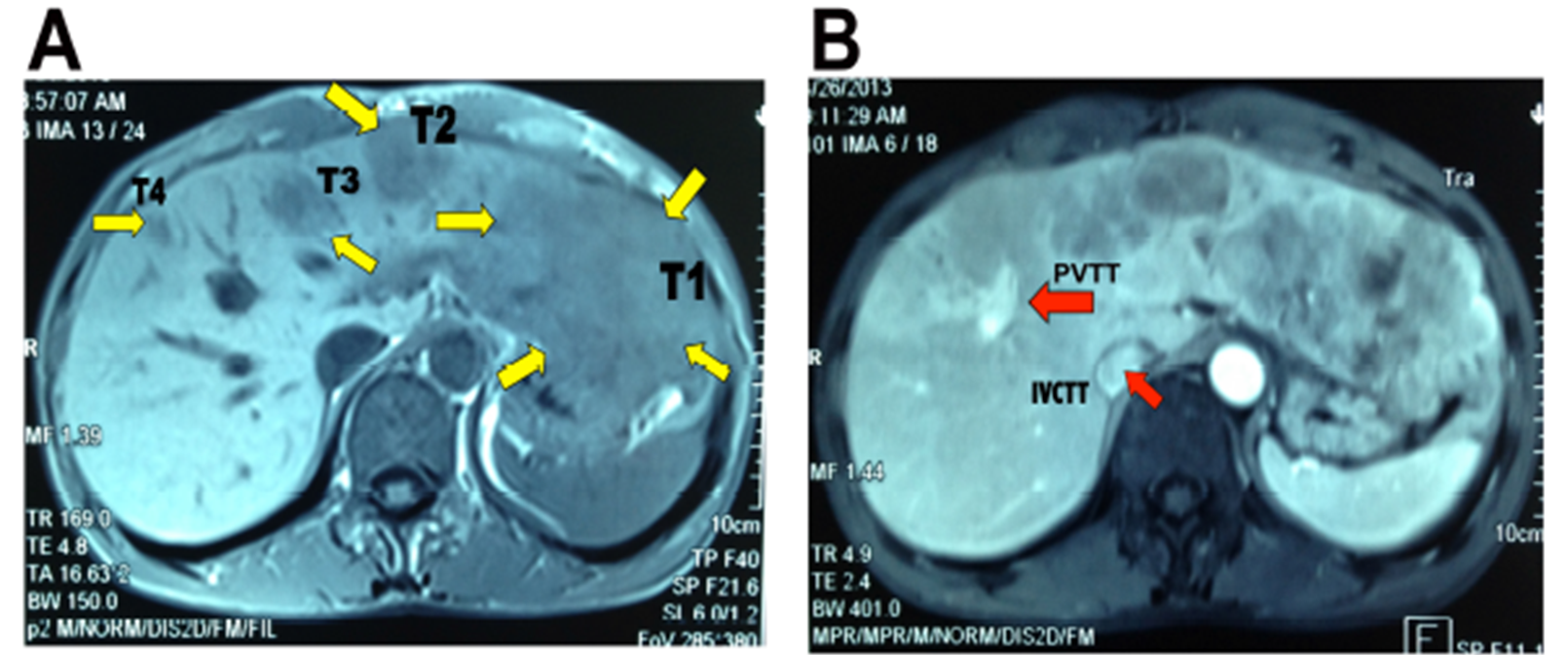

Supplement: Supplementary file 16 — Additional file 16: Fig. S1. The location of tumors and thrombi on liver. A Magnetic resonance imaging (MRI) shows a 15 cm × 10 cm larger lesion in the left hepatic lobe and multiple smaller lesions in the right hepatic lobe, all less than 3 cm in diameter. Yellow arrows indicate multiple tumor foci of various sizes. B MRI with contrast enhancement reveals tumor thrombosis involving the inferior vena cava (IVCTT), and the right portal vein branch (PVTT), indicated by the red arrows, respectively, suggesting intrahepatic and extrahepatic vascular spread of HCC. [file 12920_2022_1264_MOESM16_ESM.tiff]

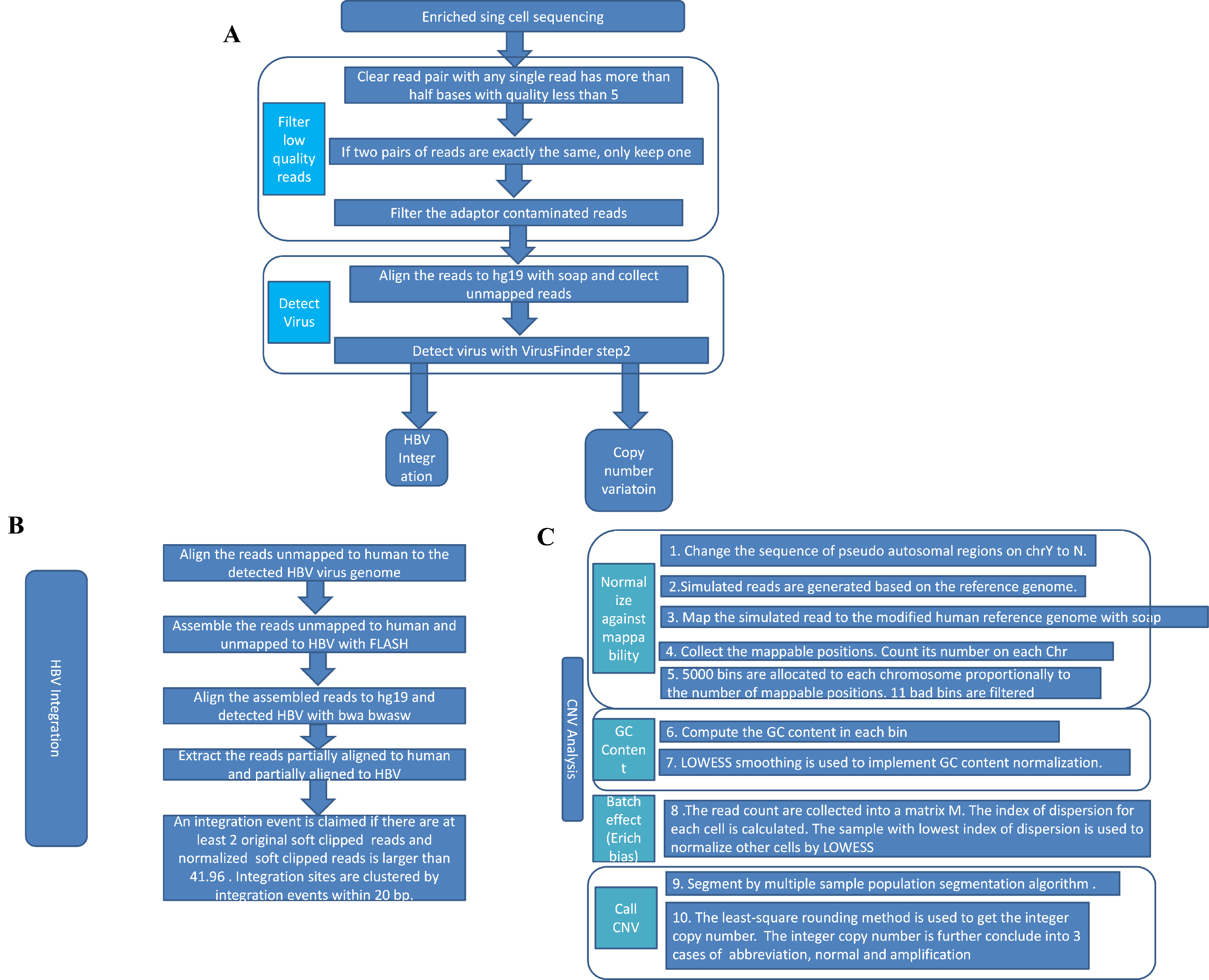

Supplement: Supplementary file 17 — Additional file 17: Fig. S2. Data analysis flow chat. A General analysis flow chat. After filtering low quality raw reads and detecting the HBV sub strain. HBV integrations and single cell CNV are called separately. B Pipeline for detecting HBV integration. C Pipeline for detecting single cell CNV. [file 12920_2022_1264_MOESM17_ESM.tiff]

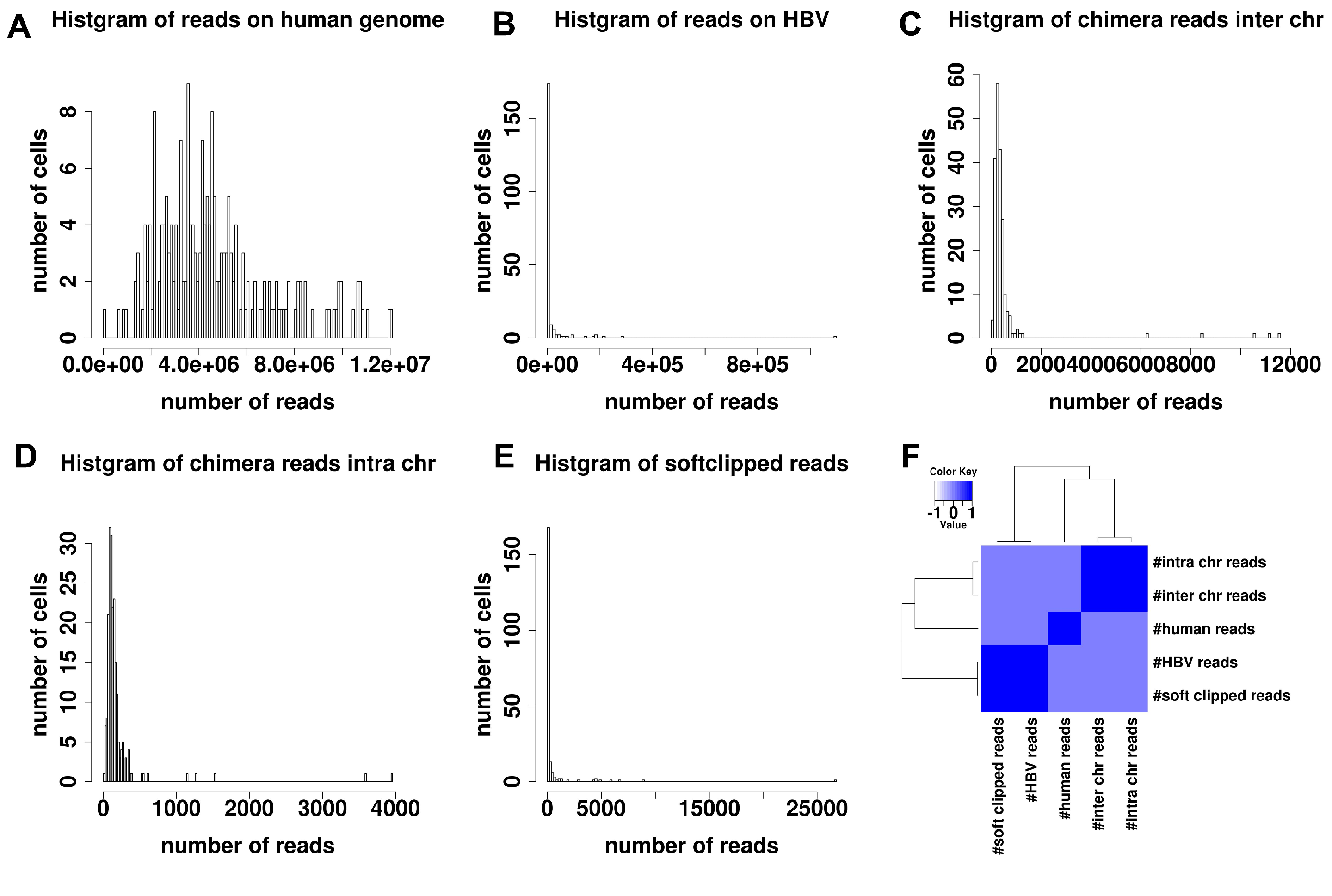

Supplement: Supplementary file 18 — Additional file 18: Fig. S3. Histograms of number of human reads (A), number of HBV reads (B), number of inter chromosome chimera reads (C), number of intra chromosome chimera reads (D), number of softclipped reads (E). The average chimera reads ratio is 0.025% which is lower than the reported chimera reads ratio of 6.19% by Tu et.al and 2%/3% by Xie’s group. F Correlation coefficients between the numbers of human reads, inter chromosome chimera reads, intra chromosome chimera reads, HBV integrations, and HBV reads. Numbers of chimera reads for inter and intra chromosome are highly correlated. Numbers of chimera reads are not correlated with number of reads on HBV, number of soft clipped reads and number of reads on human. Numbers of reads on HBV and soft clipped reads are correlated. [file 12920_2022_1264_MOESM18_ESM.tiff]

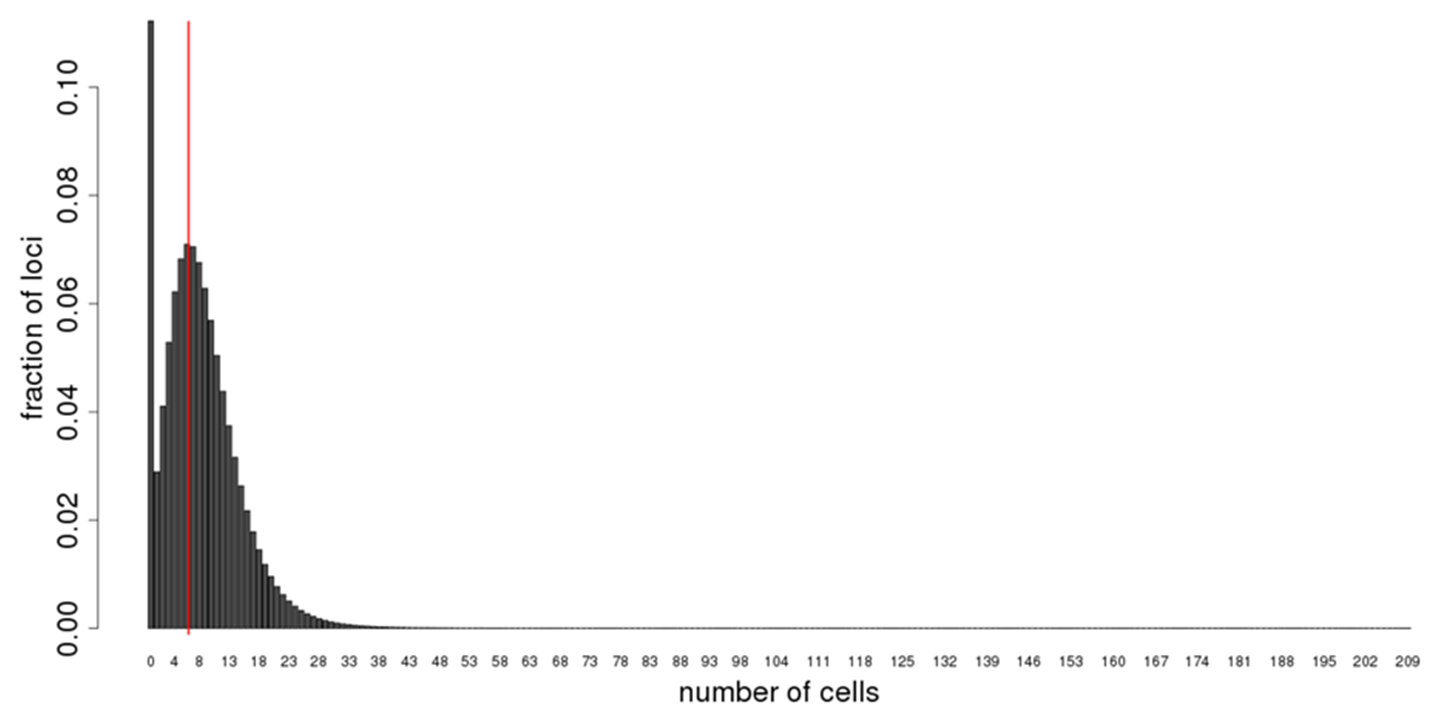

Supplement: Supplementary file 19 — Additional file 19: Fig. S4. Distribution of number of cells with reads covering the each loci. Red line indicates the mean. Each bin corresponds to the fraction of human genome is successfully sequenced in a number of cells. If the reads distribute randomly on human genome, the distribution follows Poisson distribution. [file 12920_2022_1264_MOESM19_ESM.tiff]

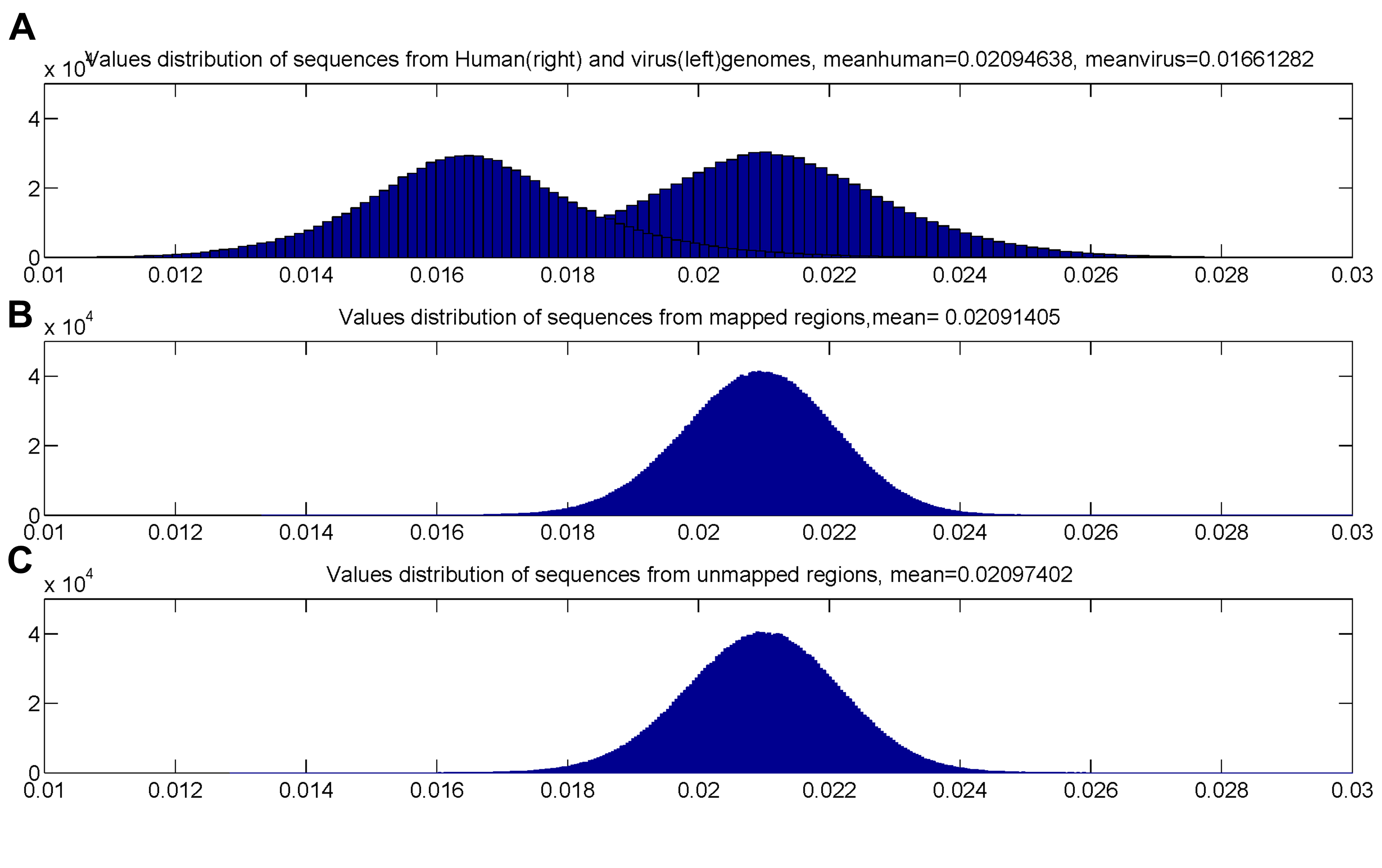

Supplement: Supplementary file 20 — Additional file 20: Fig. S5. A Compare HBV sequence and human genome sequence with Fisher values. B Fisher values from Human mapped region. C Fisher Values from Human unmapped region. [file 12920_2022_1264_MOESM20_ESM.tiff]

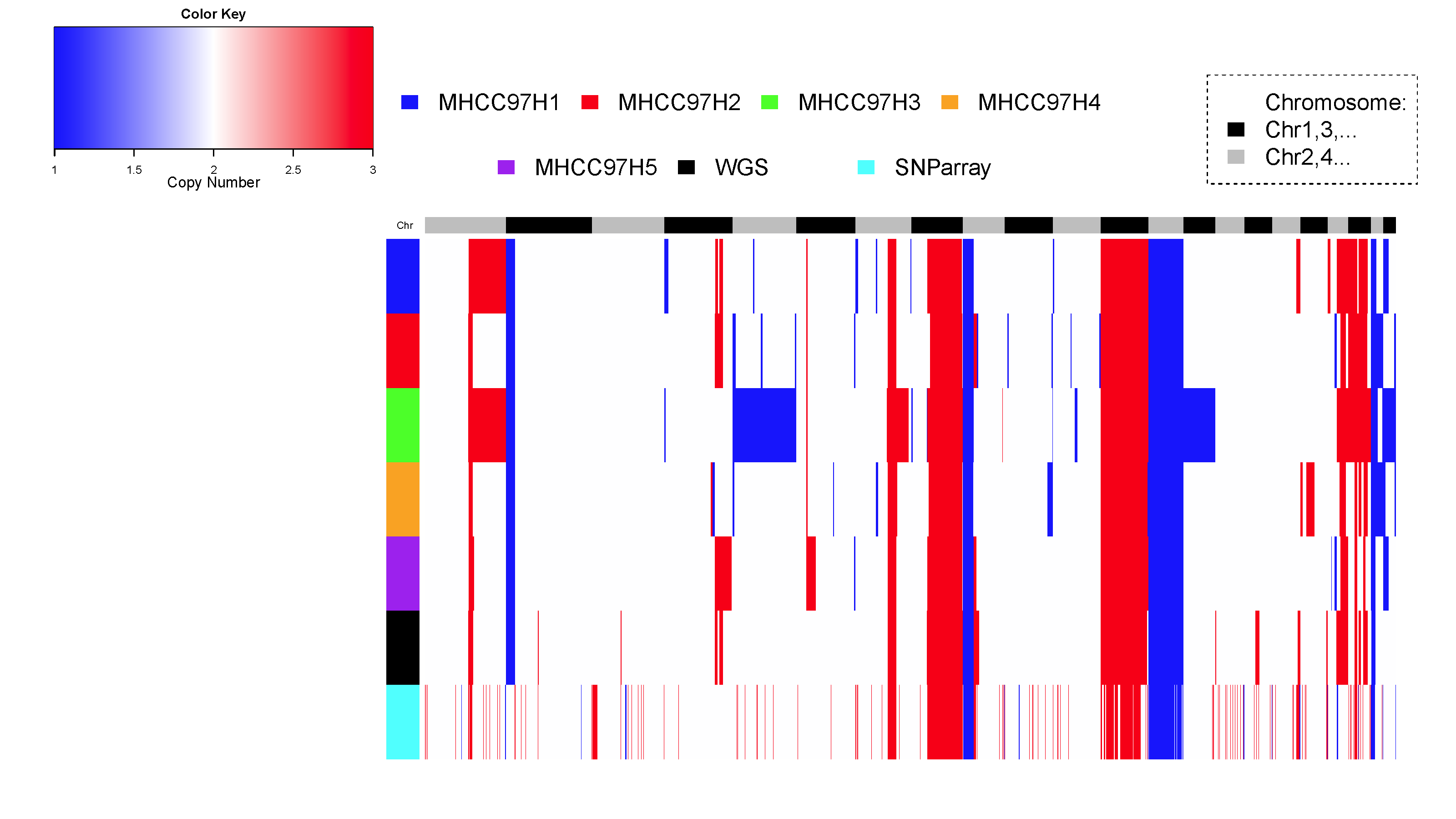

Supplement: Supplementary file 21 — Additional file 21: Fig. S6. MHCC97H’s CNV profile generated by enriched single cell sequencing, whole genome sequencing and SNParray. [file 12920_2022_1264_MOESM21_ESM.tiff]

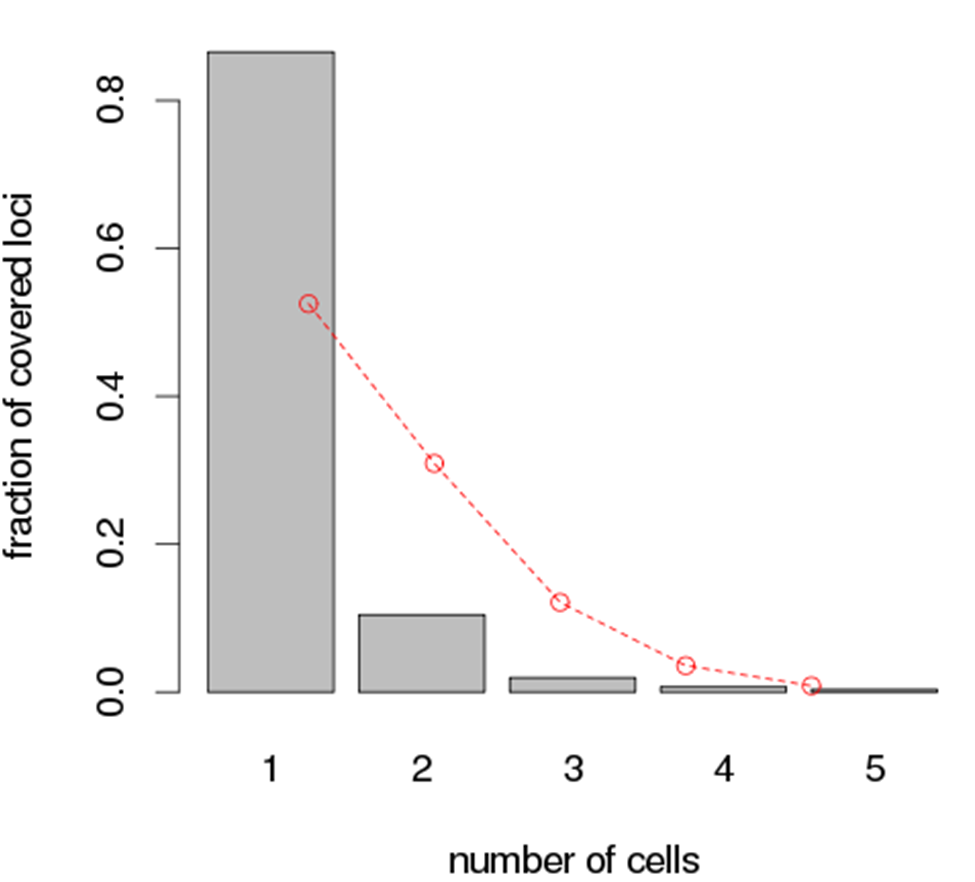

Supplement: Supplementary file 22 — Additional file 22: Fig. S7. Distribution of number of cells with reads covering the each loci for MHCC97H. Each bin corresponds to the fraction of human genome is successfully sequenced in a number of cells. If the reads distribute randomly on human genome, the distribution follows Poisson distribution. Chi-square test against Poisson distribution producing p-value 0.98. [file 12920_2022_1264_MOESM22_ESM.tiff]

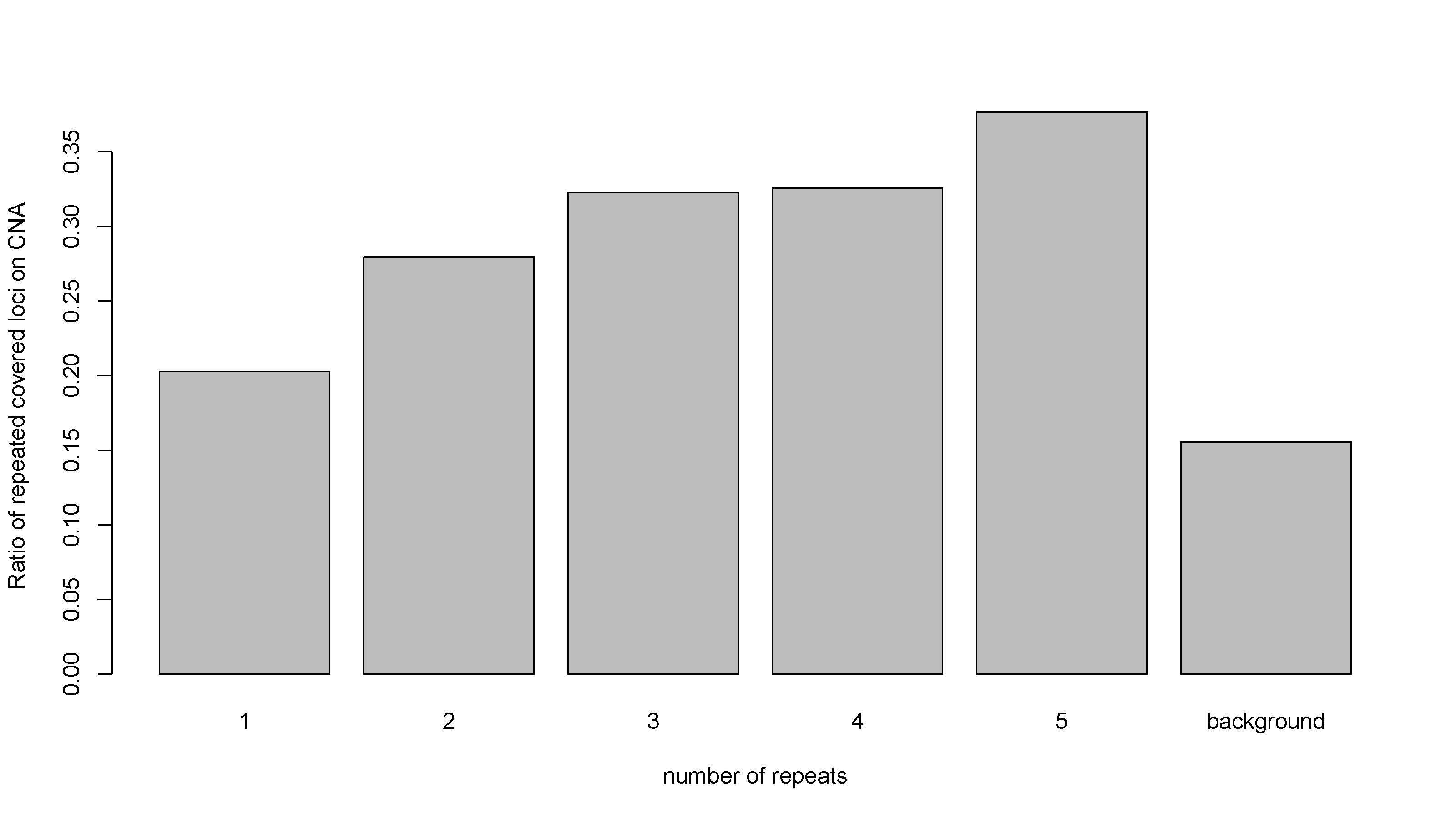

Supplement: Supplementary file 23 — Additional file 23: Fig. S8. Distribution of repeatedly covered loci across the copy number amplified region called from Whole genome sequence data for MHCC97H. [file 12920_2022_1264_MOESM23_ESM.tiff]

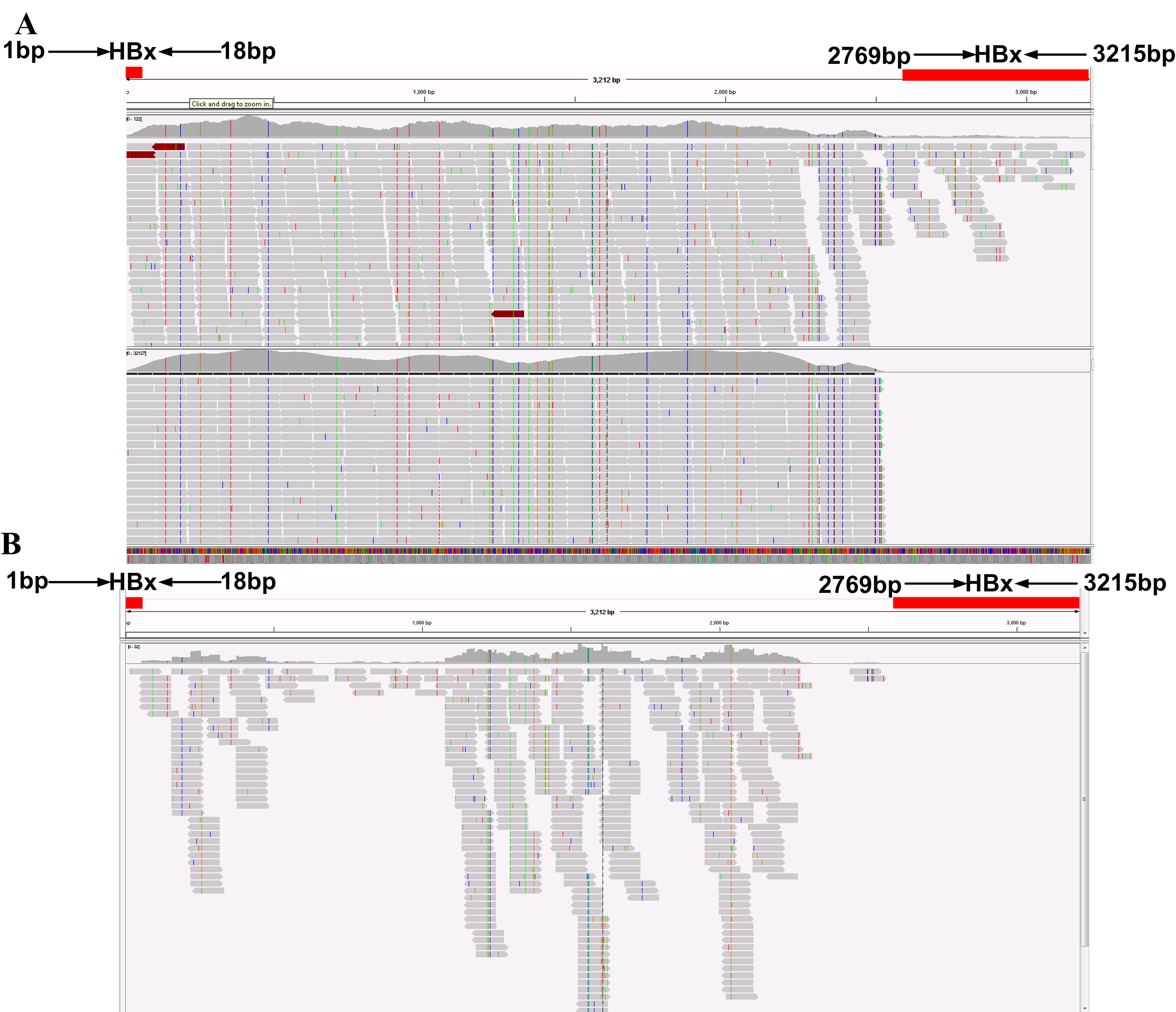

Supplement: Supplementary file 25 — Additional file 25: Fig. S10. A HBV reads pileup results for an example cell with IGV. The reference genome is G247-B3. HBx-protein region is labeled as red. B HBV reads pileup results comparing between tumor tissues and adjacent normal tissues. The upper panel is for all the HBV reads in adjacent normal tissues. The lower panel is for all the HBV reads in tumor tissues. [file 12920_2022_1264_MOESM25_ESM.tiff]

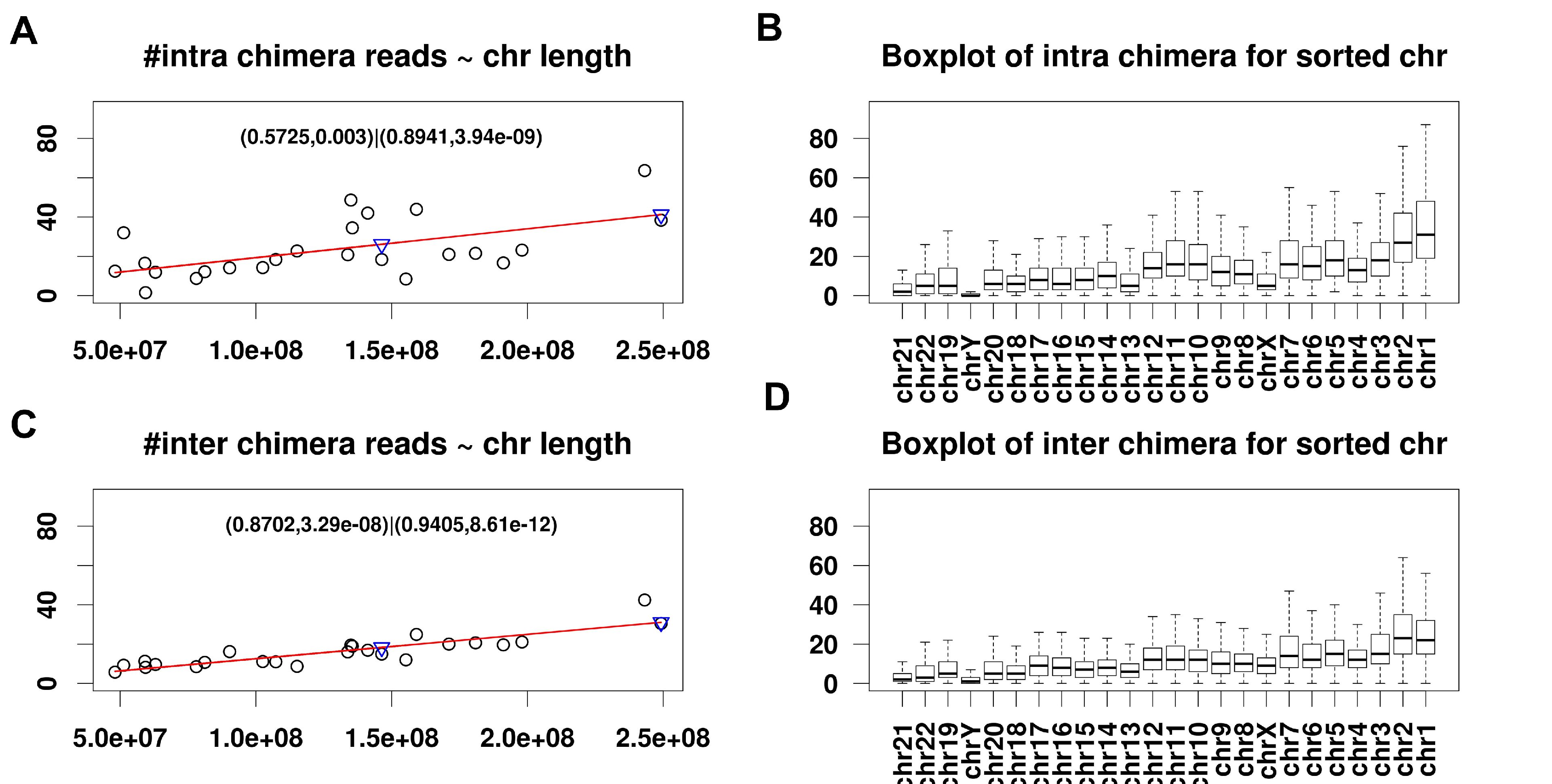

Supplement: Supplementary file 26 — Additional file 26: Fig. S11. Linear correlation between inter chromosome chimera reads, intra chromosome chimera reads and length of chromosomes. Scatter plots (A, C) and boxplot (B, D) of number of chimera reads and length of chromosome for both inter and intra chromosome cases. The blue triangles indicate Chr1 and Chr8. The numbers in A and C are (correlation between chromosomes’ length and mean # of chimera reads | p-value) and (correlation between chromosomes’ length and median # of chimera reads | p-value). The correlations between numbers of chimera reads and length of chromosome are significant. [file 12920_2022_1264_MOESM26_ESM.tiff]

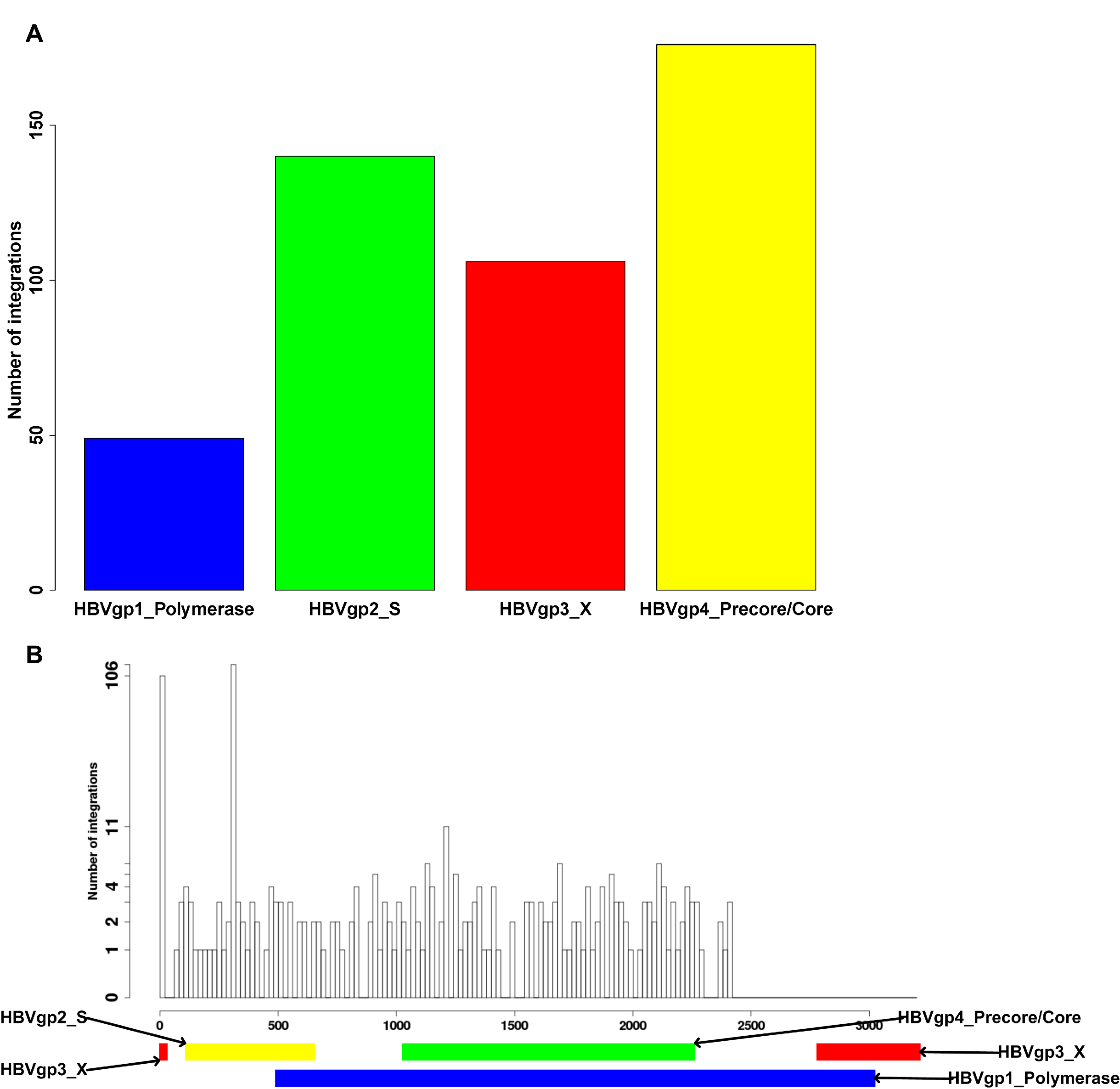

Supplement: Supplementary file 27 — Additional file 27: Fig. S12. A Distribution of HBV integrations across HBV proteins of P, S, X, C. HBV integrations are located on S, C and X. B Distribution of HBV integrations across HBV genome. [file 12920_2022_1264_MOESM27_ESM.tiff]

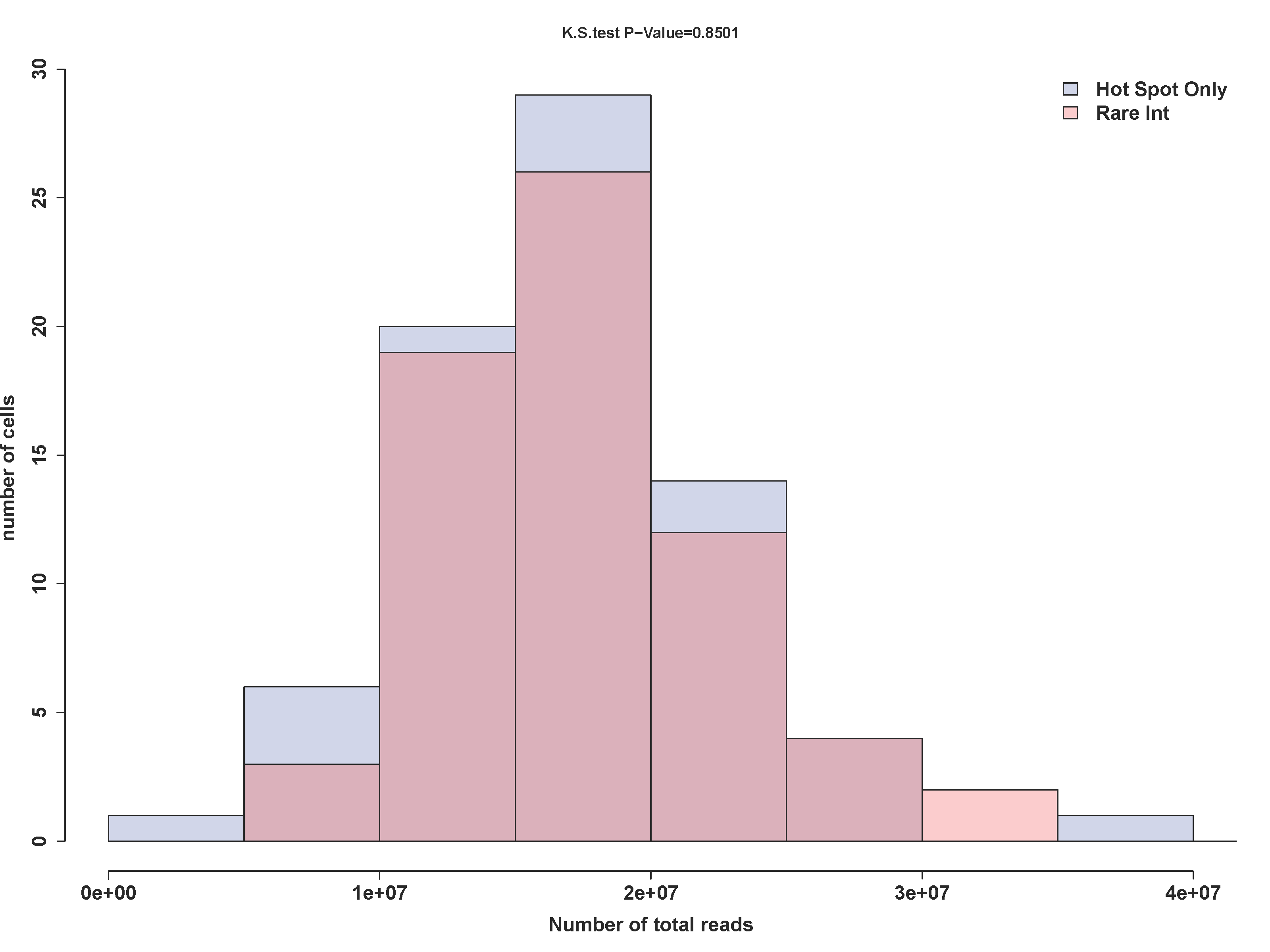

Supplement: Supplementary file 28 — Additional file 28: Fig. S13. Compare the read throughput of the two clustered sets of cells from Fig. 2C. Histograms of reads throughput from these two sets of cells are almost overlapped. K.S. test shows no significant difference between these two distributions. The set of cells carrying extra integrations other than hot spot integrations are not benefit from higher throughput of reads. [file 12920_2022_1264_MOESM28_ESM.tiff]

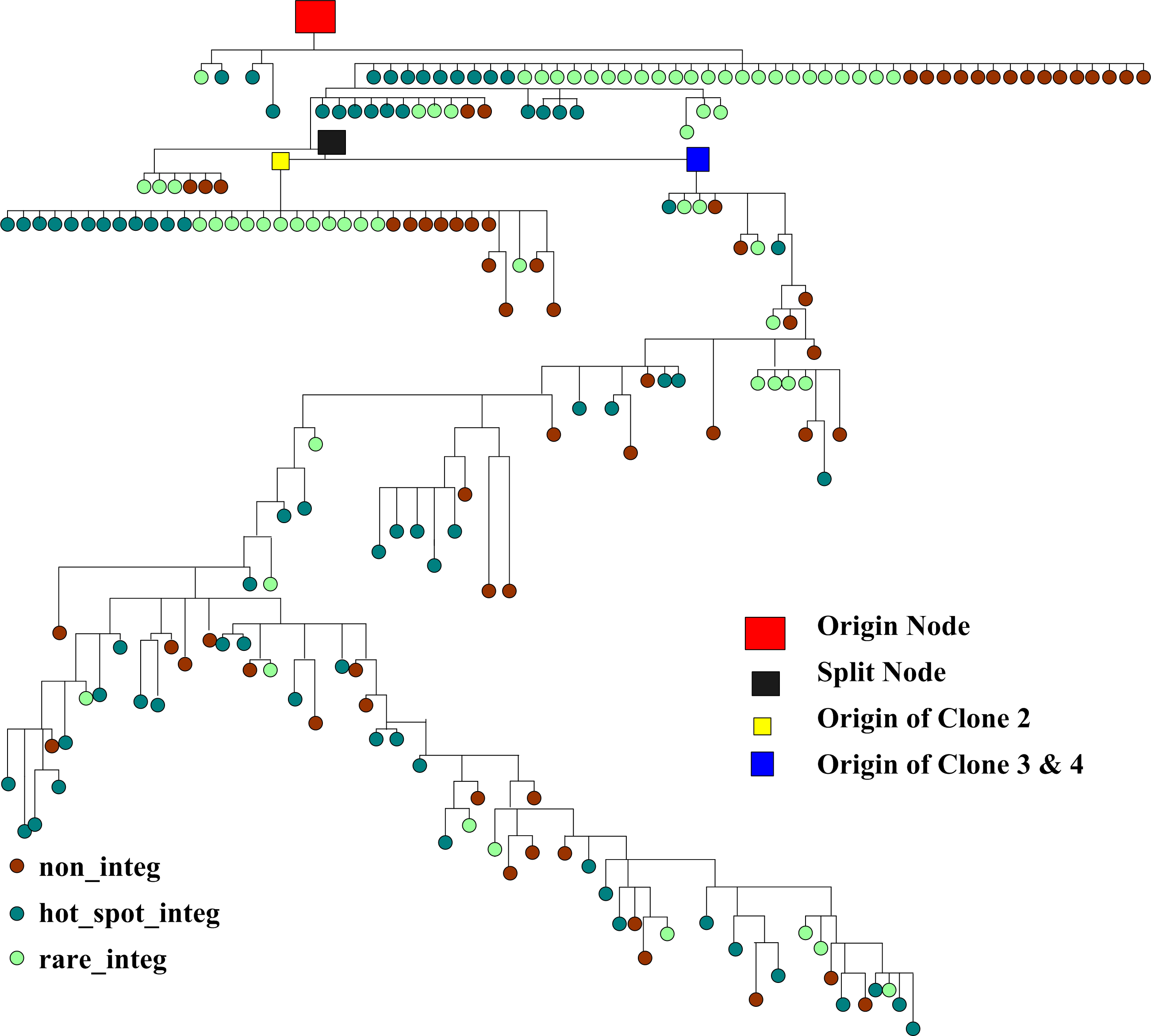

Supplement: Supplementary file 29 — Additional file 29: Fig. S14. Labeling the phylogenetic tree in Fig. 4A by carrying only hot spot integrations, extra rare integrations and no integrations. We can find that with dynamic clonal evolution. The rate of rare integration is becoming less and less. [file 12920_2022_1264_MOESM29_ESM.tiff]

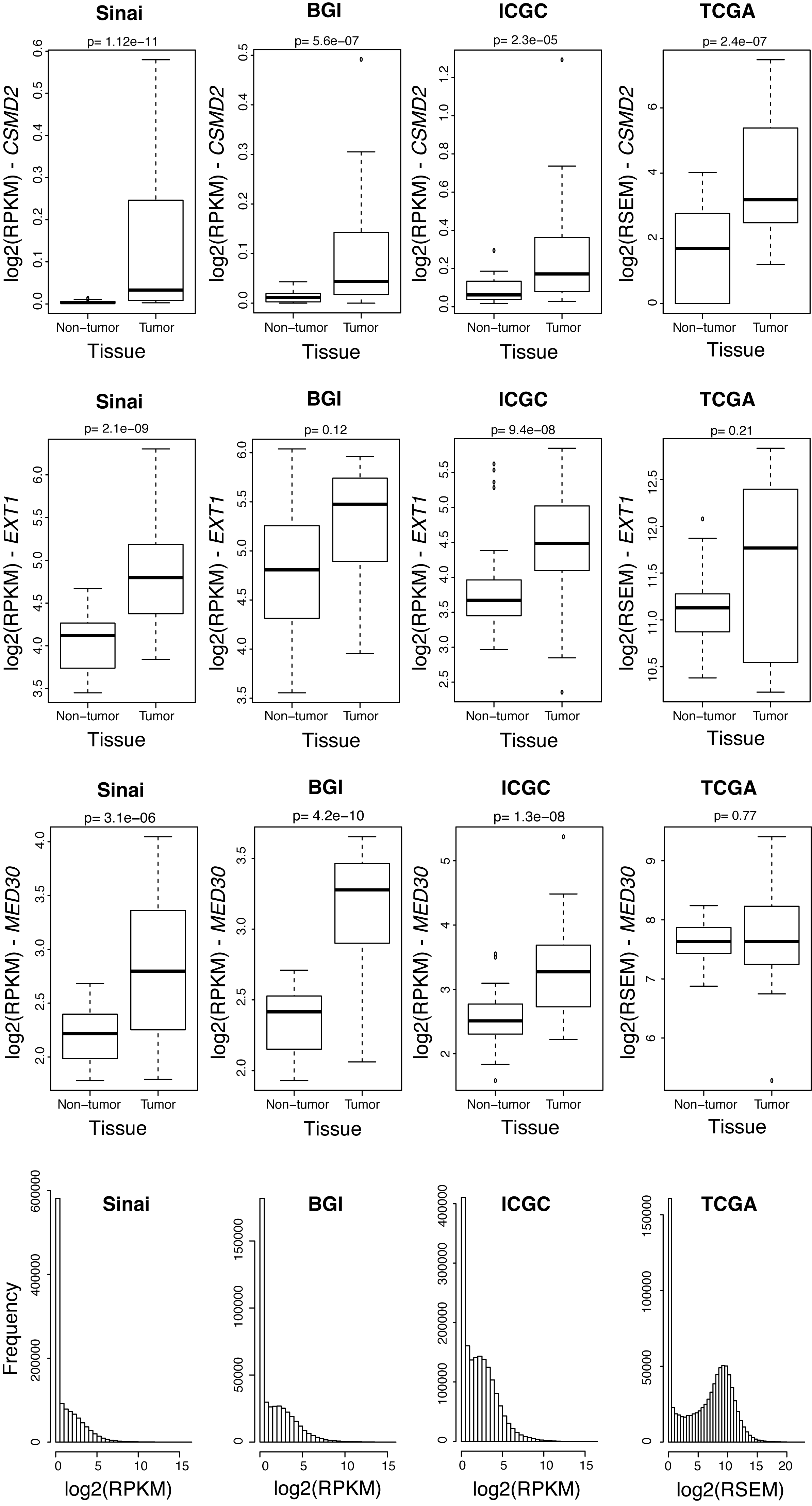

Supplement: Supplementary file 30 — Additional file 30: Fig. S15. Expression of the hot spot genes from ICGC and TCGA. Hot spot genes CSMD2, MED30, and EXT1 are find expressed significantly higher in tumor samples then adjacent normal samples. [file 12920_2022_1264_MOESM30_ESM.tiff]

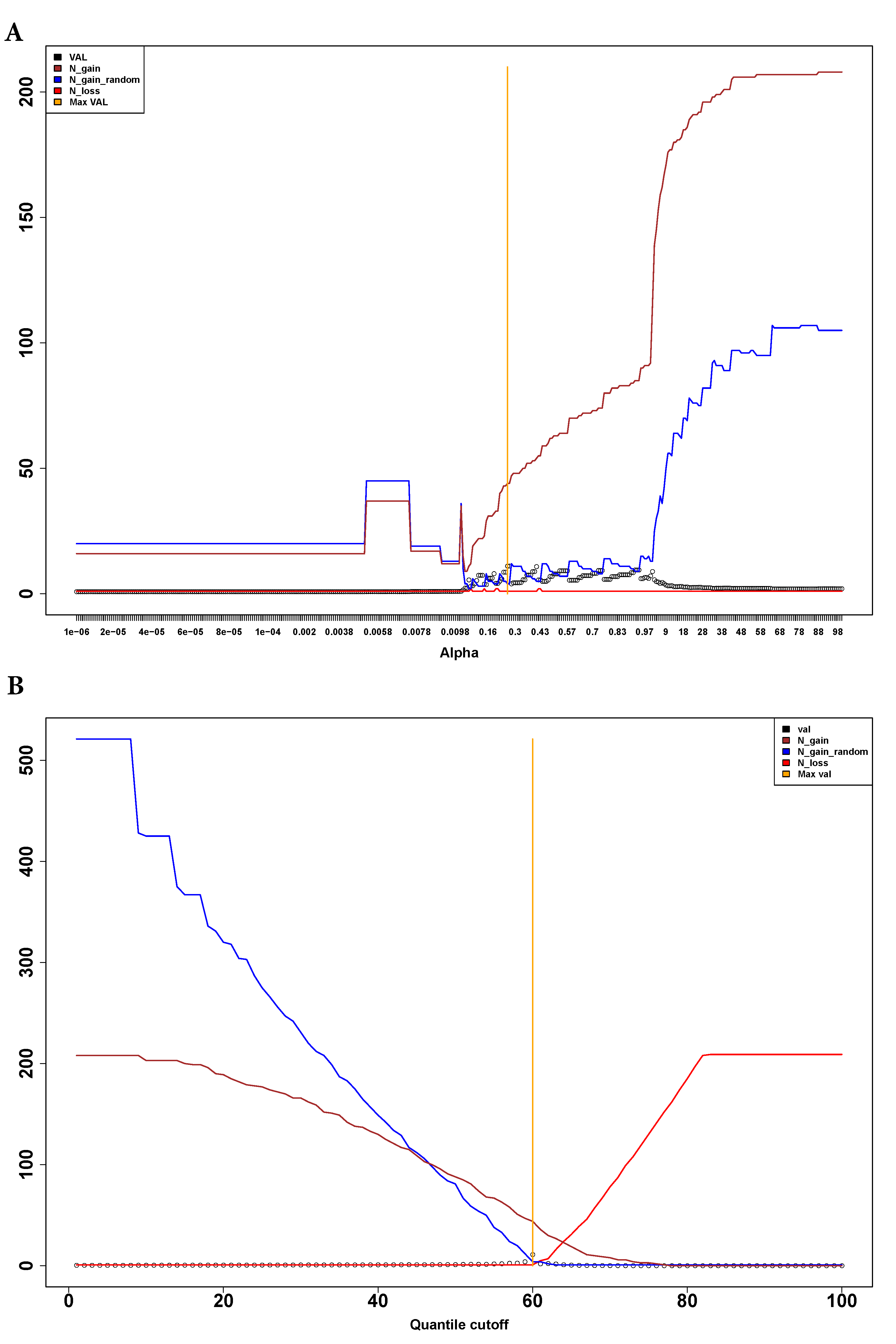

Supplement: Supplementary file 31 — Additional file 31: Fig. S16. A Find the best tuning parameter for the pseudo count and weight adjustment. B Select the best cutoff for the selected best tuning parameter. [file 12920_2022_1264_MOESM31_ESM.tiff]

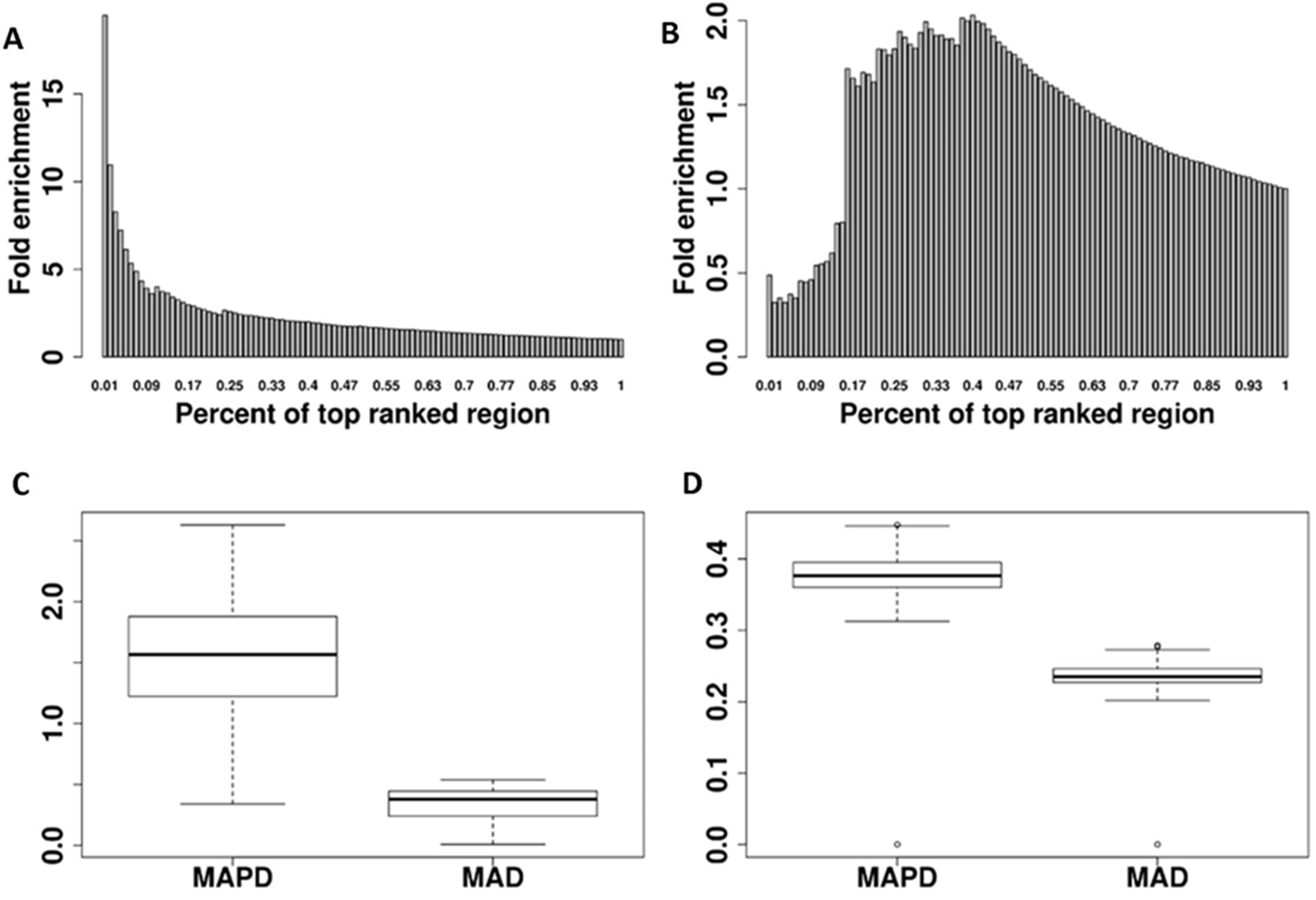

Supplement: Supplementary file 32 — Additional file 32: Fig. S17. Quality of Bin's read count correction. A Fold enrichment of top x% bins carrying HBV integration before correction. Bins are sorted by the number of reads mapped in the bin. B Fold enrichment of top % bins carrying HBV integration after correction. Bins are sorted by corrected reads. C MAPD and MAD before batch effect correction. D MAPD and MAD after batch effect correction. [file 12920_2022_1264_MOESM32_ESM.tiff]

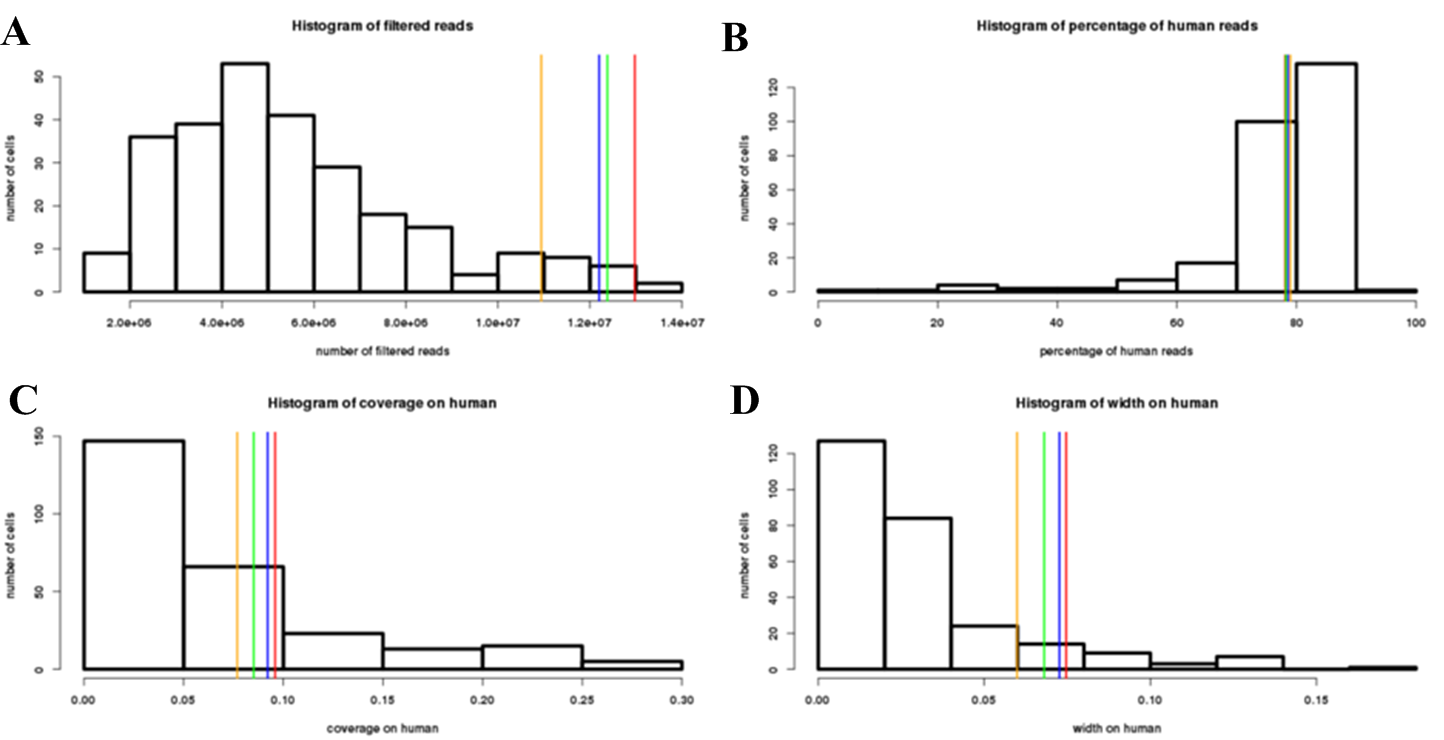

Supplement: Supplementary file 33 — Additional file 33: Fig. S18. Comparison of the number reads between normal bulk tissues and tumor single cells. Histogram shows the distribution for tumor single cells while vertical color lines show the corresponding quantity of normal control tissue. A Comparison of numbers of filtered reads; B comparison of percentage of reads mapped to human genome; C comparison of coverage on human genome; D comparison of width on human genome. [file 12920_2022_1264_MOESM33_ESM.tiff]

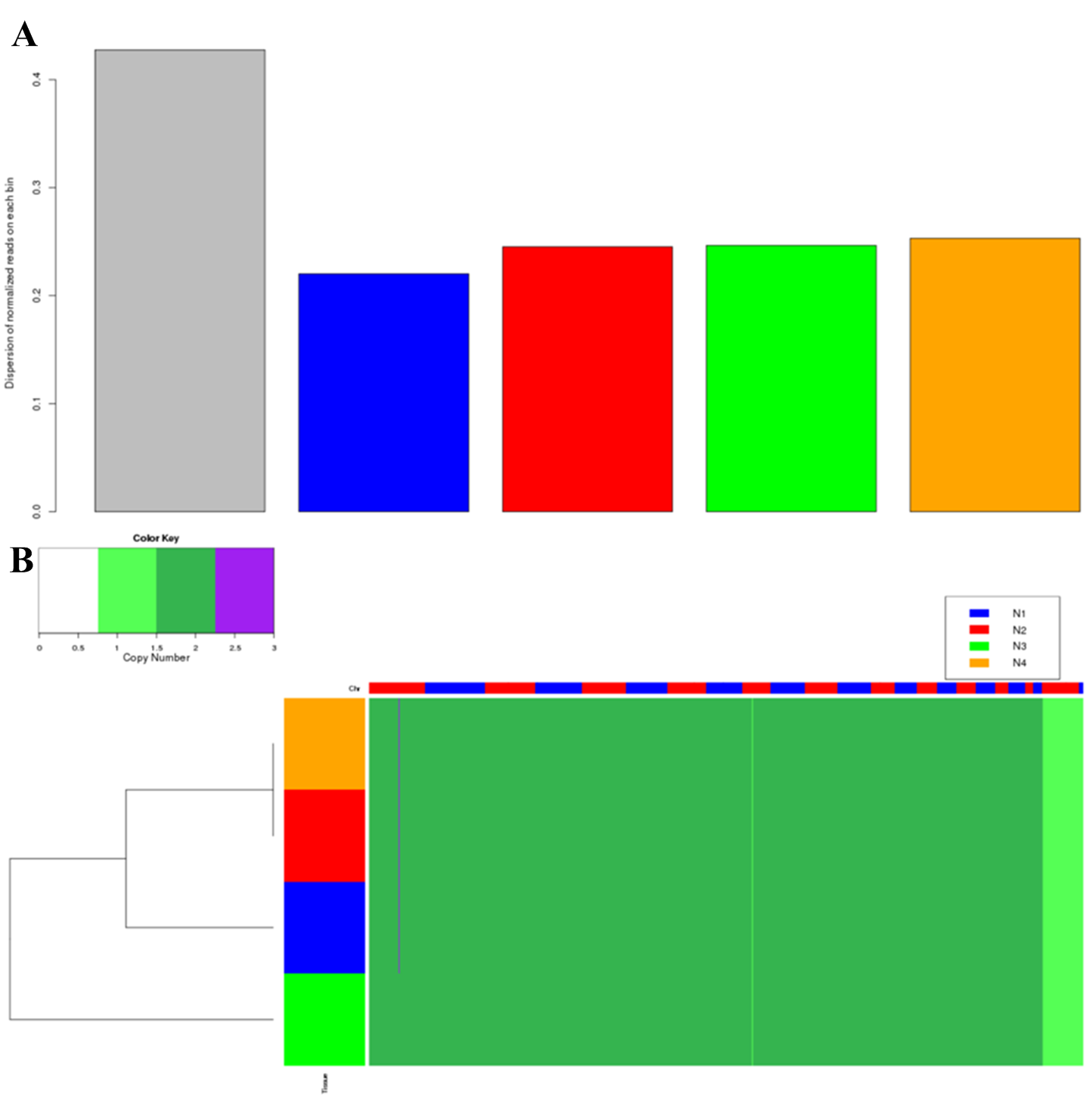

Supplement: Supplementary file 34 — Additional file 34: Fig. S19. A Comparison dispersion of binned reads count after mappability and GC content correction between the smallest one in single tumor cells and the four normal control tissue. B CNV results on normal tissues. [file 12920_2022_1264_MOESM34_ESM.tiff]
